# Sequence specificity despite intrinsic disorder: How a disease-associated Val/Met polymorphism rearranges tertiary interactions in a long disordered protein

**DOI:** 10.1371/journal.pcbi.1007390

**Published:** 2019-10-18

**Authors:** Ruchi Lohia, Reza Salari, Grace Brannigan

**Affiliations:** 1 Center for Computational and Integrative Biology, Rutgers University, Camden, New Jersey, United States of America; 2 Department of Physics, Rutgers University, Camden, New Jersey, United States of America; Max Planck Institute for Biophysical Chemistry, GERMANY

## Abstract

The role of electrostatic interactions and mutations that change charge states in intrinsically disordered proteins (IDPs) is well-established, but many disease-associated mutations in IDPs are charge-neutral. The Val66Met single nucleotide polymorphism (SNP) in precursor brain-derived neurotrophic factor (BDNF) is one of the earliest SNPs to be associated with neuropsychiatric disorders, and the underlying molecular mechanism is unknown. Here we report on over 250 *μ*s of fully-atomistic, explicit solvent, temperature replica-exchange molecular dynamics (MD) simulations of the 91 residue BDNF prodomain, for both the V66 and M66 sequence. The simulations were able to correctly reproduce the location of both local and non-local secondary structure changes due to the Val66Met mutation, when compared with NMR spectroscopy. We find that the change in local structure is mediated via entropic and sequence specific effects. We developed a hierarchical sequence-based framework for analysis and conceptualization, which first identifies “blobs” of 4-15 residues representing local globular regions or linkers. We use this framework within a novel test for enrichment of higher-order (tertiary) structure in disordered proteins; the size and shape of each blob is extracted from MD simulation of the real protein (RP), and used to parameterize a self-avoiding heterogenous polymer (SAHP). The SAHP version of the BDNF prodomain suggested a protein segmented into three regions, with a central long, highly disordered polyampholyte linker separating two globular regions. This effective segmentation was also observed in full simulations of the RP, but the Val66Met substitution significantly increased interactions across the linker, as well as the number of participating residues. The Val66Met substitution replaces *β*-bridging between V66 and V94 (on either side of the linker) with specific side-chain interactions between M66 and M95. The protein backbone in the vicinity of M95 is then free to form *β*-bridges with residues 31-41 near the N-terminus, which condenses the protein. A significant role for Met/Met interactions is consistent with previously-observed non-local effects of the Val66Met SNP, as well as established interactions between the Met66 sequence and a Met-rich receptor that initiates neuronal growth cone retraction.

## Author summary

Intrinsically disordered proteins are proteins that have no well-defined structure in at least one functional form. Mutations in one amino acid may still affect their function significantly, especially in subtle ways with cumulative adverse effects on health. Here we report on molecular dynamics simulations of a protein that is critical for neuronal health throughout adulthood (brain-derived neurotrophic factor). We investigate the effects of a mutation carried by 30% of human population, which has been widely studied for its association with aging-related and stress-related disorders, reduced volume of the hippocampus, and variations in episodic memory. We identify a molecular mechanism in which the mutation may change the global conformations of the protein and its ability to bind to receptors.

## Introduction

The physiological significance of intrinsically disordered proteins (IDPs), which can explore a wide range of conformational ensembles in their functional form, is now well-established [1–5]. More than 33% of eukaryotic proteins contain disordered regions longer than 30 residues [3], many of which are involved in critical biological functions, including transcriptional regulation [6] and cell signaling [7–9]. Long intrinsically disordered regions are particularly abundant among cancer-associated [10] and neurodegenerative-associated proteins [11, 12].

**Fig 1:**
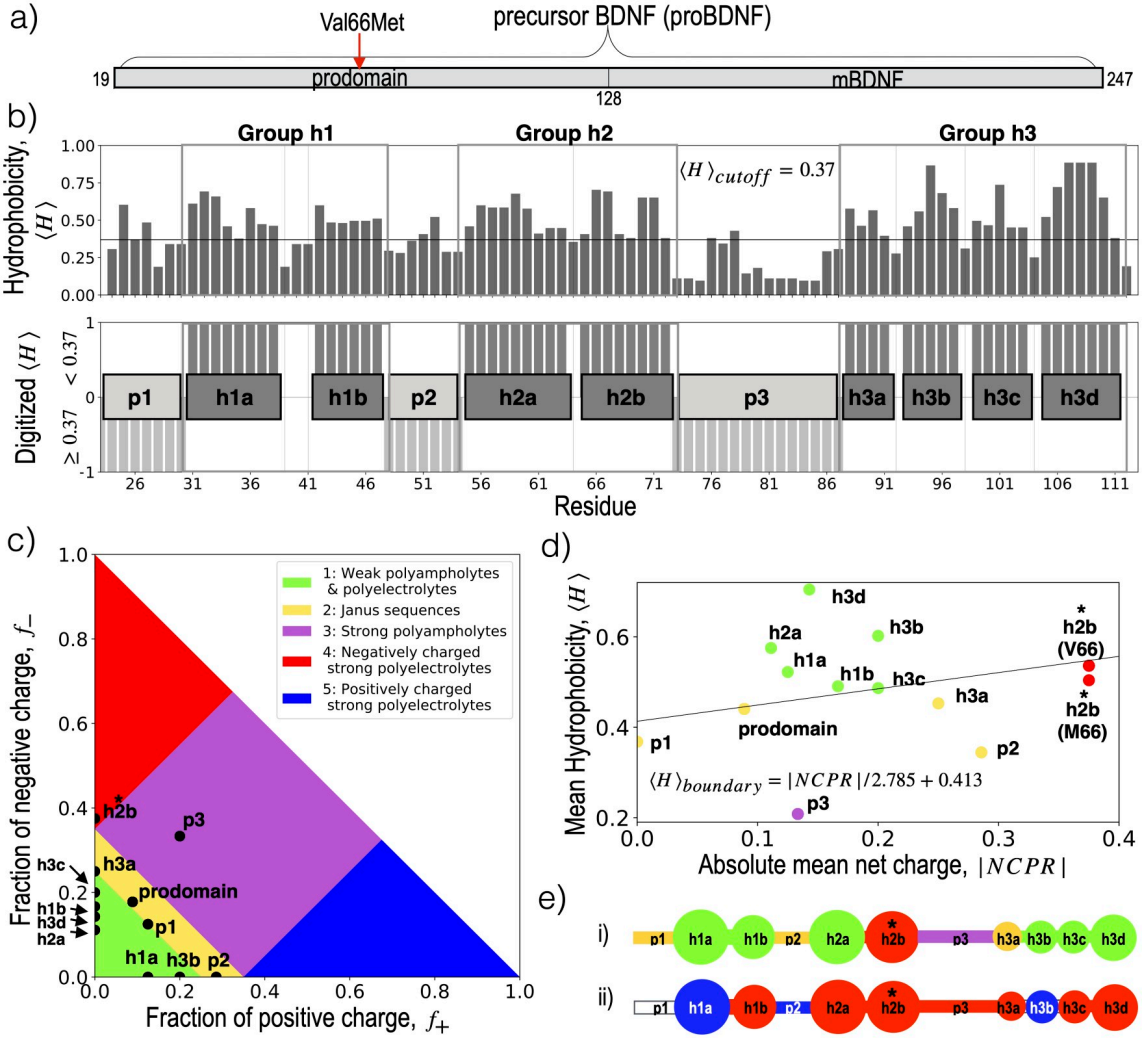
Sequence-based decomposition of the BDNF prodomain. a) The two functional domains of precursor BDNF: the disordered prodomain considered in this manuscript and the structured mature domain BDNF (mBDNF). b) The mean hydrophobicity (〈*H*〉) per residue (top), given by the Kyte-Dolittle [65] score averaged over a three residue window, and scaled to fit between 0 and 1 was digitized (bottom) according to a cutoff at 〈*H*〉 > 0.37. Four or more contiguous residues above the cutoff were identified as forming a hydrophobic blob. Eight hydrophobic “h” blobs (darkgrey) are identified along with 3 “p” blobs of low hydrophobicity (light grey). c) The diagram of IDP states proposed by Das and Pappu [21], based on fraction of positive (*f*_+_) and negative (*f*_−_) charged residues, and annotated by the location of the simulated BDNF prodomain and each blob identified in panel b. d) Location of simulated BDNF prodomain and each blob on an Uversky diagram [66] of IDPs and globular proteins, as a function of absolute net charge per residue (|*NCPR*|) and 〈*H*〉, with the boundary line between folded and disordered proteins given by the equation in the legend. e) Blobs identified in panel b, colored according to (i) the region of the Das and Pappu [21] diagram in panel c or (ii) sign of net charge, where red is negatively-charged, blue is positively-charged, and white is neutral. The blob h2b contains the Val66Met SNP and is marked with star. Additional properties of the blob sequences can be found in Table 1.

IDP amino acid sequences tend to be low-complexity [13, 14] and include numerous charged residues, often in long repeats [1, 15]. In contrast to ordered proteins, in which a complex sequence encodes a well-defined tertiary structure, an IDP sequence determines a heterogeneous conformational ensemble [16–18]. More than 35% of IDPs reported in DISPROT [19] are strong polyampholytes, and their ensemble properties can be predicted using statistical theories of polyampholytes from polymer physics and global properties of the sequence, including the fraction of charged residues and the separation of oppositely charged residues (Fig 1c) [20–23]. This role is consistent with the long-range nature of electrostatic interactions, which can affect coupling between distant residues in an otherwise disordered structure.

Although IDP sequences are low-complexity and do not encode a well-defined structure, single residue substitutions can still have functional effects that are significant for the organism [24]. More than 25% of disease-associated missense single nucelotide polymorphisms (SNPs) are found in IDPs [25]. Although detectable, the relatively subtle functional effects of these SNPs may lead to relatively weak selection pressure, whether positive or negative, allowing the mutation to persist at high frequencies within a population. Numerous structural and simulation studies [26–32] have demonstrated clear effects of single charged-residue insertion, deletion, or substitutions on conformational ensemble and aggregation of IDPs monomers. Simple electrostatic models predict that modifications of residue charge will directly affect ensemble properties [20, 26, 33, 34]. Locally, such mutations can modulate residual secondary structure preferences via forming or breaking local salt-bridges or by introducing helix breaking residues [27, 31, 35].

For IDPs with a relatively low fraction of charged residues, typical of the Janus region of the state diagram proposed by Das and Pappu [20, 21] (Fig 1c), more subtle differences among neutral amino acids play an increasingly important role in determining the ensemble. More than 40% of disease-associated IDP polymorphisms annotated in the human UniProtKB/Swiss-Prot database [36] are substitutions between two charge-neutral residues. The extent to which such substitutions in IDPs can affect non-local aspects of the conformational ensemble is uncertain; these substitutions directly affect short-range interactions, and structure-based coupling between distant residues in IDPs is expected to be weak. Nonetheless, correlations between secondary structure of distant residues has been frequently observed in IDPs [27, 37, 38]; for example, several cancer mutations in transactivation domain of tumor suppressor p53 can lead to helicity changes in residues sequentially far away from the mutation sites [27].

In structured proteins, contacts between residues distant along the sequence are reflected in the tertiary structure, but developing a framework for describing the analogous property in IDPs has not been straightforward. Among traditional structural biology techniques, NMR has been most useful for characterizing IDPs, but is frequently limited to residual secondary structure (Ref. [11, 39] and references therein). Molecular dynamics (MD) simulations have played a significant role in understanding IDP structure and dynamics [40–45], but face limitations on chain length similar to those incurred in simulations of protein folding. Most unbiased simulations have been performed in implicit solvent and/or involve chains too short to meaningfully sample contacts between residues far apart on the peptide chain. Studies of aggregation among multiple shorter monomeric IDPs [46, 47] have provided some of the most useful frameworks for considering tertiary contacts between residues that are distantly connected along the peptide backbone. Point mutations are also known to affect these contacts via differential salt-bridge and hydrogen-bonding formations, with mutations that change charge states affecting conformational ensemble via altered salt-bridge networks [46].

Many SNPs in IDPs are associated with neurological, aging-associated neurodegenerative, or psychiatric disorders; despite an exponential increase in the amount of available genetic data, identifying the genetic origins of such disorders has proven remarkably challenging, with few variants identified as replicable predictors of disease. One of the earliest identified variants is the Val66Met SNP (rs6265) in precursor brain-derived neurotrophic factor (BDNF), a signaling protein that retains a critical role in neurogenesis and synaptogenesis throughout adulthood [48, 49] (Fig 1a). It has been implicated in maintenance of the hippocampus [50, 51], orientation selectivity in the visual system [52–54] and the mechanism underlying action of numerous antidepressants [55, 56], including rapidly acting low-dose ketamine [57]. An extensive library of genome-wide association studies (GWAS) have repeatedly identified the Val66Met SNP as reducing hippocampal volume and episodic memory, as well as predicting increased susceptibility to neuropsychiatric disorders including schizophrenia, bipolar, and unipolar depression, but associations have been inconsistent and population dependent [57–61].

Difficulties in obtaining unambiguous disease associations at the precursor BDNF Val66Met SNP using GWAS are paralleled by challenges in characterizing its effects on the properties of the BDNF prodomain using structural techniques. A crystal structure of a homologous neurotrophic factor in complex with a shared receptor revealed a well-defined volume corresponding to the prodomain, but lacked resolvable density [62]. The prodomain sequence falls in the Janus sequence region in the phase diagram proposed by Das and Pappu [20, 21].

It was subsequently revealed that the cleaved prodomains are found in monomeric states *in vivo*, and the M66 (but not V66) form binds to SorCS2 (sortilin-related VPS10p domain containing receptor 2), leading to axonal growth cone retraction [63] and eliminated synapses in hippocampal neurons [64]. NMR measurements on the prodomain confirmed significant intrinsic disorder for both forms, with differential secondary structure preference around residue 66 [63]. Tertiary contact distances from NOEs were not accessible, however, and uncertainty in interpretation of the NMR signal prevented evidence of non-local effects on secondary structure from being conclusive. Additional NMR experiments implicated residue 66 in binding of M66 prodomain to SorCS2 [63].

In this work, we aimed to provide insight into the following questions: (1) What interactions drive the secondary structure change local to residue 66 observed through NMR? (2) How can we meaningfully detect tertiary interactions in a long disordered protein? (3) Do effects on tertiary interactions explain the non-local secondary structure changes previously observed through NMR? (4) How and why does the Val66Met mutation change tertiary interactions, especially as a charge-neutral mutation? To achieve these aims, we conducted unbiased fully-atomistic replica-exchange MD simulations of the 91 residue BDNF prodomain in explicit solvent, for V66 and M66 sequence.

We begin by identifying globular regions, or blobs, within the protein using a sequence-based approach based on residue hydrophobicity; this is useful for both conceptualizing the long disordered protein in the absence of a well-defined topology, as well as focusing the analysis. We then compare our simulation results with previous NMR results of Anastasia et al. [63] and discuss the effects of the Val66Met SNP on residual secondary structure. We propose and apply an approach for decoupling short-range structural correlations from long-range structural correlations, by comparison with a simplified polymer model parameterized from the MD trajectories. We then discuss the effect of the Val66Met SNP on the network of correlated *β* strands between distant residues, illustrating how effects of the mutation propagate to tertiary contacts in which the mutation is not involved. Finally, we identify individual residue sidechains that drive the observed effects on this network. Our results suggest an important and previously-unconsidered role for specific Met-Met interactions in transducing the effects of the BDNF Val66Met SNP, and confirm the presence of weak but long-range structural correlations in a disordered protein.

## Results and discussion

### Prodomain sequence decomposition

The region of the BDNF prodomain studied using NMR [63], and simulated here, is 91 residues long. Conceptualization of long structured proteins relies heavily on the consecutive secondary structure elements that form the protein’s topology, allowing for a coarse cartoon-style representation. No such approach for constructing an IDP topology has been available. Our original motivation for identifying globular segments in the sequence was to improve statistical power in analyzing contacts, but we found the resulting topological description to be broadly useful for interpretation of results. We thus present this conceptual tool upfront for clarity.

To avoid ambiguity, we restrict use of the term “domain” to refer to the two major BDNF domains (mature domain and prodomain), and instead specify three levels of hierarchy below the domain level: the prodomain contains multiple “regions”, regions contain “groups”, and groups contain “blobs”. Blobs and groups were identified by sequence alone, as described in *Methods*, while regions were identified by Monte Carlo simulation of a simplified polymer representing the blobs.

The sequence-analysis approach outlined in *Methods* divides the sequence into alternating groups, classified as either hydrophobic (h groups) or non-hydrophobic (p groups). The prodomain is composed of six such groups, notated as p1-h1-p2-h2-p3-h3 from N-terminus to C-terminus. The h groups are further divided into blobs (Fig 1b), indexed with a letter. Each hydrophobic group contains two to four blobs: h1 contains h1a and h1b, h2 contains h2a and h2b, and h3 contains h3a, h3b, h3c, and h3d. We denote multiple consecutive blobs within a group by multiple letters: h3ab indicates the stretch of residues between the beginning of blob h3a and the end of blob of h3b. Each p group consists of just one blob. The results in *Regions of tertiary enrichment* led us to further designate Region I (containing p1 through h2), Region II (comprised of p3) and Region III (comprised of h3).

Since each blob sequence has its own properties (Table 1), this process also suggested a new, more tractable conceptualization of the long, disordered BDNF prodomain. Each blob can be analyzed individually according to Das and Pappu metrics [21] (Fig 1c) or Uversky metrics [66] (Fig 1d), while several other sequence properties of each blob are shown in Table 1. The Das and Pappu phase diagram [21] predicts the compactness of IDPs based on their fraction of positively (*f*_+_) and negatively (*f*_−_) charged residues (Fig 1c). Hydrophobic blobs h2b and h3a lie in the strong polyelectrolyte and Janus sequence region respectively. All the remaining hydrophobic blobs are classified as weak polyampholytes and, as isolated peptides, would be predicted to have compact globule conformations to shield hydrophobic residues [21]. Linker blobs p1 and p2 also lie in the Janus sequence region, while blob p3 lies in the strong polyampholyte region. The charge distribution parameter *κ* [21] is also low for p3, which is predicted to have random coil conformations if present as an isolated peptide.

**Table 1:** Sequence based properties of hydrophobic (h) and linker (p) blobs identified in the BDNF prodomain, as shown in Fig 1.

Region	Group	Blob	N^a^	NCPR^b^	〈*H*〉^c^	FCR^d^	*f*_−_^e^	*f*_+_^f^	*κ*^g^	Sequence	R^h^	P^i^
I	p1	p1	8	0.00	0.37	0.25	0.13	0.13	0.8	EANIRGQG	2	0.00
	h1	h1a	8	0.13	0.52	0.13	0.00	0.13	1.0	GLAYPGVR	1	0.13
		h1b	6	-0.17	0.49	0.17	0.17	0.00	0.1	TLESVN	1	0.00
	p2	p2	7	0.29	0.34	0.29	0.00	0.29	0.4	GPKAGSR	2	0.14
	h2	h2a	9	-0.11	0.58	0.11	0.11	0.00	0.7	GLTSLADTF	1	0.00
		h2b(V66)	8	-0.38	0.54	0.38	0.38	0.00	0.3	HVIEELLD	4	0.00
		h2b(M66)	8	-0.38	0.50	0.38	0.38	0.00	0.3	HMIEELLD	4	0.00
II	p3	p3	15	-0.13	0.21	0.53	0.33	0.20	0.1	EDQKVRPNEENNKDA	3	0.06
III	h3	h3a	4	-0.25	0.45	0.25	0.25	0.00	N/A	DLYT	2	0.00
		h3b	5	0.20	0.60	0.20	0.00	0.20	N/A	RVMLS	1	0.00
		h3c	5	-0.20	0.49	0.20	0.20	0.00	N/A	QVPLE	1	0.20
		h3d	7	-0.14	0.70	0.14	0.14	0.00	1.0	PLLFLLE	1	0.14
V66 Seq			91	-0.09	0.44	0.26	0.18	0.09	0.2		2	0.07
M66 Seq			91	-0.09	0.44	0.26	0.18	0.09	0.2		2	0.07

^*a*^ Number of residues in the blob

^*b*^ Net charge per residue

^*c*^ Mean hydrophobicity, average of Kyte-Dolittle [65] scores for each residue in the blob scaled to fit between 0 and 1

^*d*^ Fraction of charged residues

^*e*^ Fraction of negatively charged residues

^*f*^ Fraction of positively charged residues

^*g*^ Charge distribution parameter *κ* as defined by Das and Pappu [21], calculated using CIDER [67]

^*h*^ Region in phase diagram proposed by Das and Pappu [21] (Fig 1c)

^*i*^ Fraction of Proline residues

The Uversky diagram [66] characterizes proteins as globular or intrinsically disordered based on their normalized mean hydrophobicity (〈*H*〉) and absolute net charge per residue (|*NCPR*|) (Fig 1d). The proteins falling above the boundary line are predicted to be globular proteins, while the ones below that line are predicted to be IDPs. With the exception of hydrophobic blobs h2b and h3a, all hydrophobic blobs identified here fall in the globular side of the boundary. Blobs h2b, h3a and p1 fall on the disordered side of the boundary, while p2 and p3 fall deep in the disordered side of the boundary.

The blob h2b contains V/M66, and has several unique properties among the identified blobs: 1) it is located at the sequence midpoint 2) it is the only strong polyelectrolyte blob 3) it has the strongest NCPR (-0.38) among all the blobs 4) its sequence is composed almost entirely of two competing residue types, yielding the uncommon mix of a highly-charged, hydrophobic blob. Considering mean hydrophobicity alone, Uversky et al. [66] found 〈*H*〉 ∼ 0.48 ± 0.03 for a set of 275 folded proteins and 〈*H*〉 ∼ 0.39 ± 0.05 for a set of 91 unfolded proteins. By this criteria, we would expect the h2b sequence to be folded: for V66-h2b, 〈*H*〉 ∼ 0.54, while for M66-h2b, 〈*H*〉 ∼ 0.50. The full Uversky diagram also considers NCPR, and the high NCPR pushes h2b into the IDP region of the Uversky diagram [66].

More specifically, this blob sequence (HV/MIEELLD) has hydrophobic residues at i, i-1, i+3, and i+4 separated by acidic residues at i+1 and i+2. Helix formation would thus segregate hydrophobic residues from acidic residues but would also increase the density of like-charge residues. Similar sequences are observed in the activation domains of transcription factors: a motif of alternating hydrophobic and acidic residues folds into an amphipathic helix upon binding, and the interactions between the amphipathic helix and the binding partner are mediated by hydrophobic residues, not charged residues [68–72]. Staller et al. [72] have earlier reported that in the disordered acidic activation domain of Gcn4, the acidic residues keep key hydrophobic residues exposed to solvent and binding partners.

The blob h3a is a unique hydrophobic Janus blob with high NCPR. Janus sequences have intermediate compositional biases and their conformations are context dependent [20, 21]. The SNP blob h2b and the Janus blob h3a are separated by the long (15 residue) strong polyampholyte linker p3, which has well mixed charge (*κ* = 0.1). The blobs h1a and h3b are positively charged and all the remaining hydrophobic blobs are negatively charged (Fig 1e).

### Comparison of experimental observables and their computational analogues

NMR spectroscopy [63] has previously confirmed the intrinsic disorder of the prodomain. Many of the common force-field and water model combinations used for MD simulations are optimized for folded proteins, and are not recommended for IDPs [73, 74]. Piana et al. [74] showed that several such force-field and water model combinations produced substantially more compact disordered states when compared with experiments. In order to predict accurate ensembles of the prodomain, we tested several force-field and water model combinations, optimized for IDPs, including a03sbws [75, 76] with Tip4p/2005 [77], a99sbws [76, 78] with Tip4p/2005 [77], a99sb*-ildn-q [78, 79] with Tip4p-D [74] and c36m [80] with Tip3p [81] on 30 residue fragments of the V66 prodomain using temperature replica-exchange molecular dynamics (T-REMD), further described in S1 Table. To minimize the effects of loss of long-range contacts in the 30 residue fragment, only Δ*δ*C_*α*_ were compared; Δ*δ*C_*β*_ is more dependent on *β*-pairing within the sequence. Among all the force-fields tested, only a03sbws with Tip4p/2005 and a99sb-ildn with Tip3p yielded significant deviations from NMR. The three remaining force-fields compared reasonably well (Δ*δ*C_*α*_ RMSD <0.5 ppm) (S1 Fig, S1 Table). This is also consistent with the force-field comparison study by Robustelli et al. [82], which observed that for IDPs with little or no secondary structure, both c36m and a99sb*-ildn-q with Tip4p-D yielded the best agreement with experimental NMR measurements.

**Fig 2:**
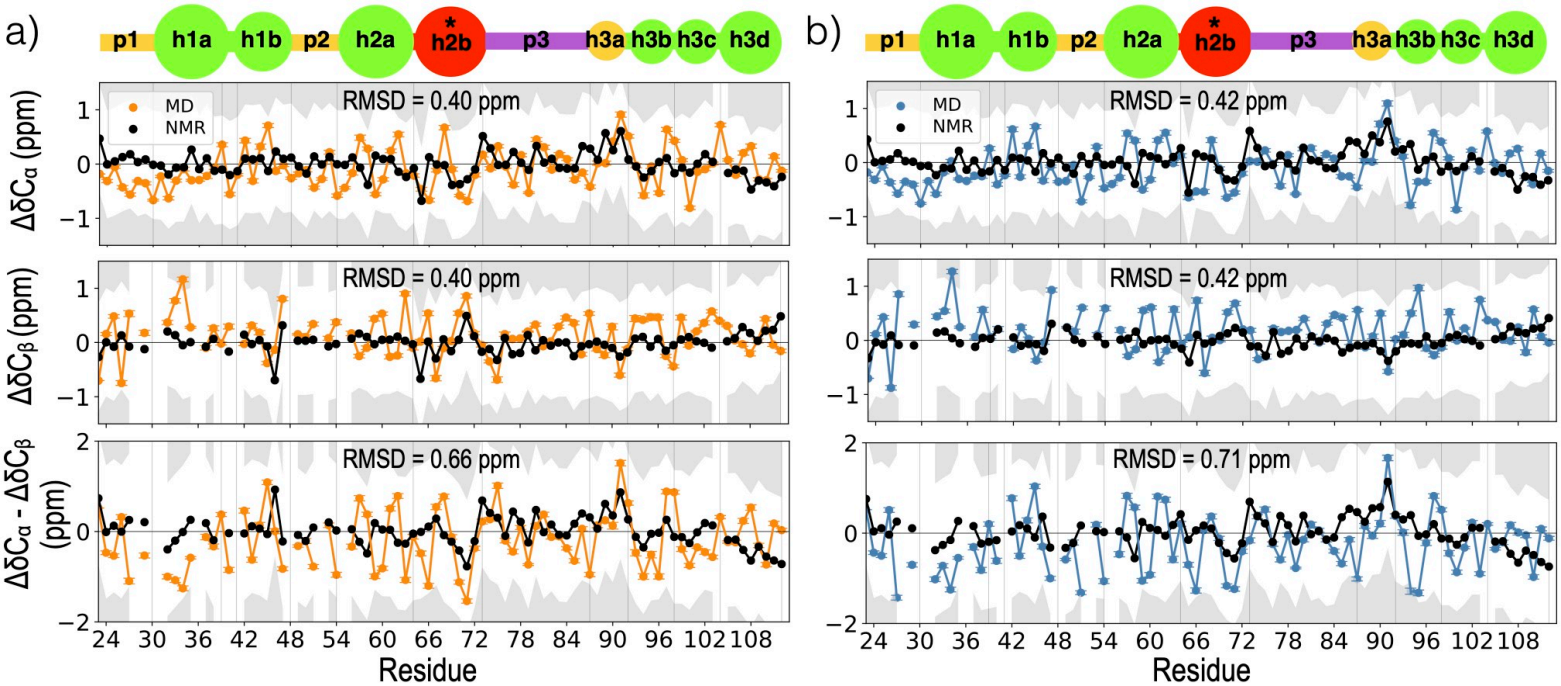
Comparison of MD and NMR observables. a) Δ*δ*C_*α*_ (top), Δ*δ*C_*β*_ (middle), Δ*δC*_*α*_-Δ*δ*C_*β*_ (bottom) values from NMR at 280K (black lines) [63] and MD at 300K for the V66 (a) and M66 (b) sequences. The gray region represents a discrepancy of more than 1 ppm from NMR secondary chemical shifts. Root-mean-squared deviation (RMSD) represents the deviation between the NMR and MD values. Error at each residue is calculated as the standard error in the mean, where *n* = 1088 is the product of the total number of replicas simulated and the average number of roundtrips per replica. Panels are annotated by a blob representation of the prodomain, as in Fig 1e(i); vertical grey lines in each panel represent the blob boundaries.

The a99sb*-ildn-q/Tip4p-D force-field was used for the full prodomain MD simulations further described in *Methods*. Fig 2 shows the C_*α*_ and C_*β*_ secondary chemical shifts calculated from the full-length simulations using SPARTA+ [83] (further described in Methods) and compares them with the NMR secondary chemical shifts obtained from Anastasia et al. [63] for the V66 and M66 sequences. We obtain good agreement with NMR secondary chemical shifts: the discrepancy at each residue is <0.7 ppm, which is less than the individual SPARTA+ prediction uncertainties of ∼ 1 ppm [83].

Comparison of the simulated hydrodynamic radii (*R*_*h*_) generated from MD and from NMR/SAXS is an additional useful validation measure [73, 84, 85]. *R*_*h*_ was calculated from the trajectory using Hydropro [86] (further described in Methods). Mean hydrodynamic radii of both the V66 (〈*R*_*h*,*V*66_〉 = 2.202 ± 0.006 nm) and M66 (〈*R*_*h*,*M*66_〉 = 2.187 ± 0.005 nm) sequences are in excellent agreement with the experimental values from NMR diffusion measurements [63] (*R*_*h*,*V*66_ = 2.24 ± 0.1 nm and *R*_*h*,*M*66_ = 2.20 ± 0.1 nm) (Convergence and distribution are discussed in Methods). Error bars for simulation results represent statistical uncertainty and do not include the additional systematic uncertainty of about 5% or 0.1 nm associated with use of Hydropro [86]. Although the M66 sequence is slightly more compact, the distributions of both *R*_*h*_ and the simulated radius of gyration (*R*_*g*_) demonstrate that the V66 and M66 sequence populate closely overlapping ensembles (See Methods). Our results support previous reports [74, 82] on the importance of pairing a99sb*-ildn-q with the Tip4p-D water model in simulations of disordered proteins; prodomain simulations with Tip3P resulted in significantly more compact ensembles.

### Effects of Val66Met on local and non-local secondary structure

**Fig 3:**
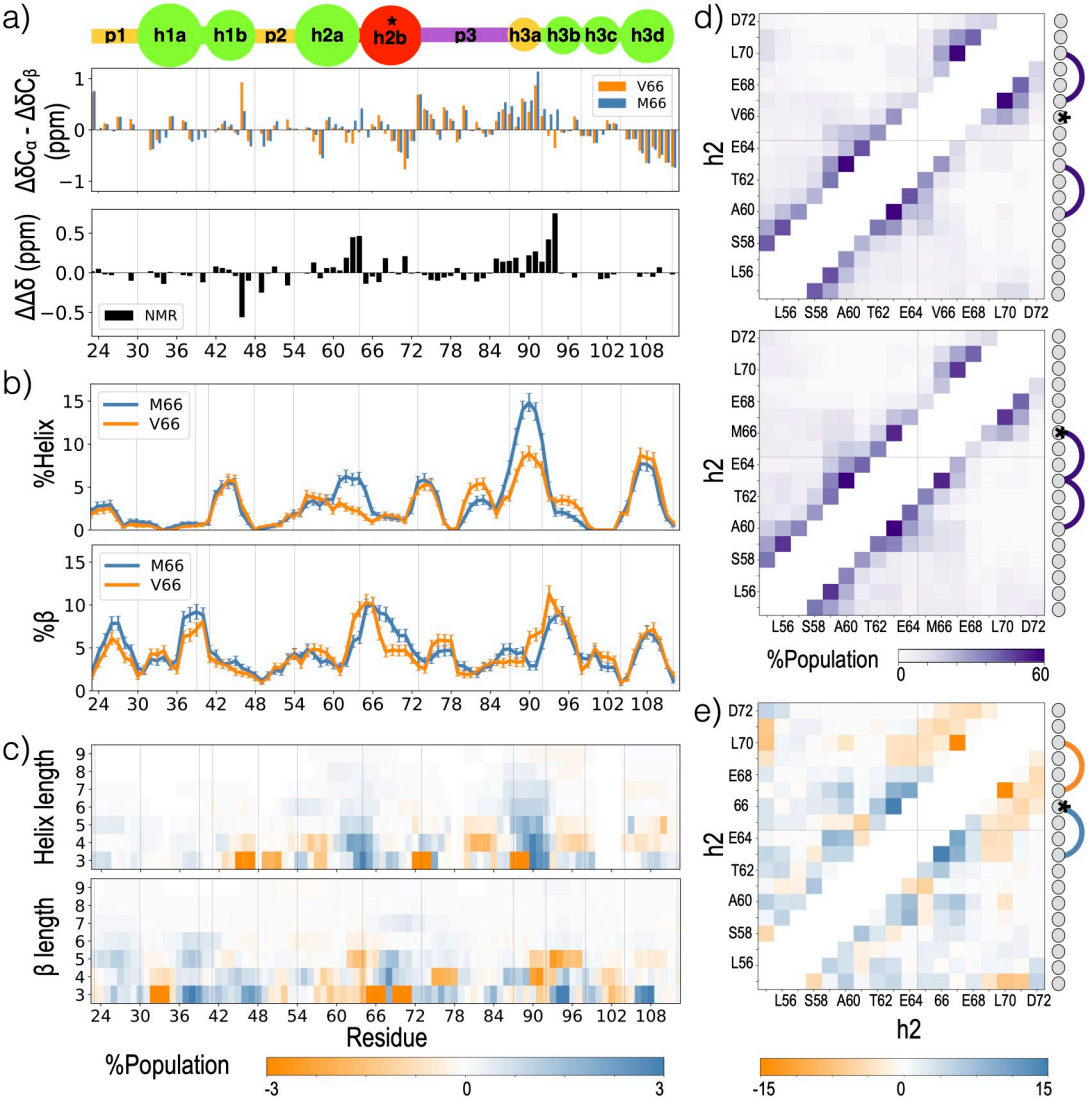
Effects of Val66Met on secondary structure. a) Δ*δC*_*α*_-Δ*δ*C_*β*_ values for the V66 and M66 sequences from NMR [63]. Values on top are equivalent to the two NMR curves shown in Fig 2 (bottom panel), while the difference between the two curves is shown at the bottom. b) Helix (top) or *β* (bottom) propensity for each simulated residue of the 300K replica, defined as the probability of a given residue being part of a sequence of four or more consecutive residues whose dihedral angles place them in the helical (left) region or *β* (right) region of the Ramachandran map (further described in Methods). Errors represent standard error of a Bernoulli trial with *n* samples, where *n* = 1088 is the product of the total number replicas and the average number of roundtrips per replica. c) Difference (M66-V66) between probabilities of secondary structure formation of a given length, for helix (top) and *β* (bottom). Panels are annotated by a blob representation of the prodomain, as in Fig 1e(i); vertical grey lines in each panel represent the blob boundaries. d) Contact probability for each residue pair within the h2 group for V66 (top) and M66 (bottom) sequences. Each residue in group h2 is annotated with a circle representation and contacts found in at least 50% of the frames are represented with an edge. e) Difference (M66-V66) between the contact probabilities shown in panel d. Contacts with a population difference of at least 15% between the V66 and M66 sequences are represented by an edge.

Anastasia et al. [63] reported an increase in helical tendency for the M66 sequence within blob h2 and h3ab and an increase in *β* tendency within blob h3b in the V66 sequence (Fig 3a). Consistent with these NMR experiments [63], the M66 sequence demonstrates an increased tendency of forming helices within blob h2 and h3a relative to the same blobs in the V66 sequence (Fig 3b). Comparing the length of secondary structure formed at each residue (Fig 3c) reveals an even stronger effect of the mutation that would not have been detectable via NMR: Val66Met consistently increases the frequency of long helices formed within group h2.

In general, C^*β*^-branched amino acids, such as valine, have more restricted side-chain rotamers in helical conformation when compared with non-C^*β*^-branched amino acids. Creamer et. al. [87] ranked the entropic cost of helix formation for apolar side chains using simulations of an (Ala)_8_ sequence with the guest amino acid at the center, and reported a higher entropic cost of helix formation for valine when compared with methionine. In our simulations, the likelihood that V66 will be in a short helix decreases with temperature, while the opposite effect is observed for the M66 (S2 Fig). These trends are consistent with an increased entropic cost for helix formation at V66 relative to M66.

The helical structure within group h2 in M66 sequence is also stabilized by local sequence, including the favorable interaction between M66 (i) and F63 (i-3). MD simulations have previously shown the stability of a sulfur-aromatic contacts in a model helix [88]. Fig 3d shows the residue level contact map within group h2. For the M66 sequence, M66 (i) more frequently contacts F63 (i-3) than any other residue within the blob: M66-F63 is formed 2.6 times as often as M66 (i)-E69 (i+3) (Fig 3d). We find that the largest change in intrablob contacts from V66 to M66 is the gain of contact at M66-F63 (1.7 times as often in M66 when compared with V66) followed by loss of contact at I67 (i+1)-L70 (i+4) (0.75 times as often in M66 when compared with V66) (Fig 3e). This is also consistent with a previously identified role for Met-Phe interactions [88–91].

While the effects of the Val66Met mutation on secondary structure in the blob which contains residue 66 (h2b) are not unexpected, we also observed an effect on secondary structure in group h1 and blobs h3a and h3b within group h3. As shown in Fig 3c, the increased frequency of long helices for blob h3a in the M66 sequence is comparable to the increase in blob h2b. We consider the possible tertiary origins of the non-local effects on secondary structure in *Effects of Val66Met on the *β*-pairing network*.

### Regions of tertiary enrichment

The potential number of residue-residue contacts in the prodomain is 91 × 90/2 ∼ 4000, and each contact is formed infrequently (S3 and S4 Figs). Detecting significant differences for numerous weak signals is statistically prohibitive, even given the long simulations presented here. Dividing the sequence into blobs based on sequence hydrophobicity (Fig 1b), as described in *Methods*, helps address this analysis challenge. Such coarse-graining reduces the number of potential contacts to 11 × 10/2 = 55, while increasing the likelihood that any given contact will be formed.

**Fig 4:**
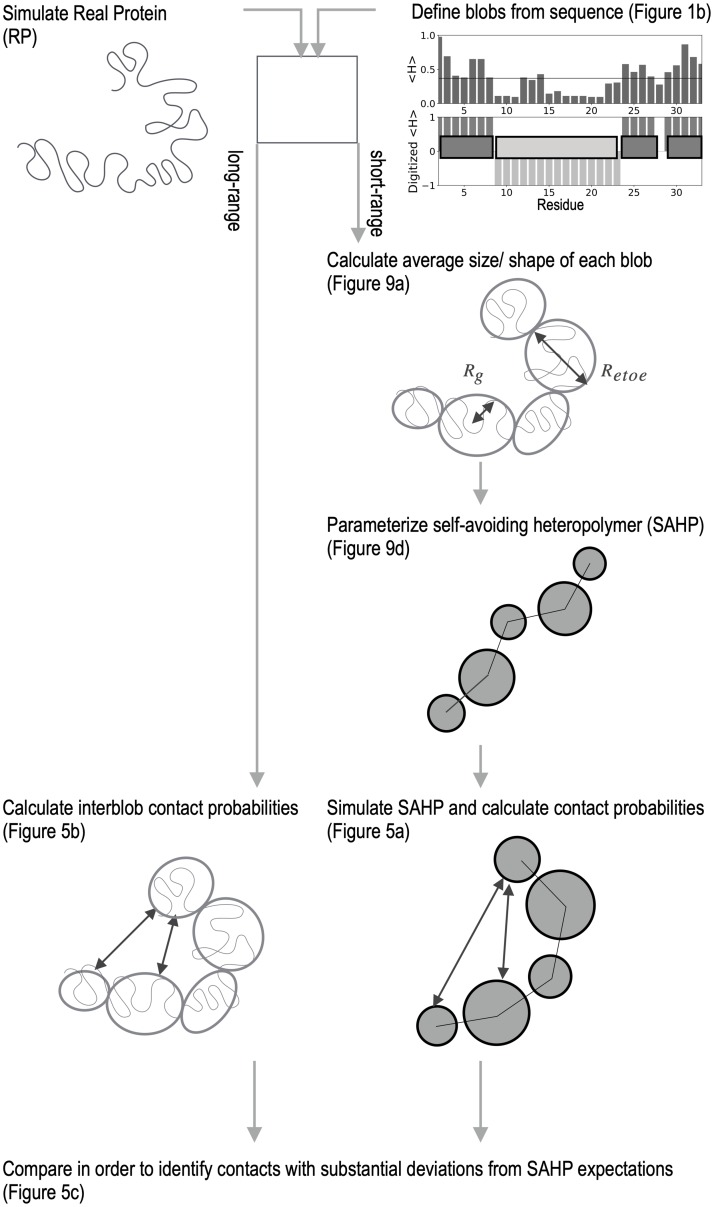
Detection of tertiary enrichment. To decouple short-range and long-range structural correlations, this work grouped segments of the protein into blobs using sequence, and then compared contacts between the blobs to those expected for an analogous self-avoiding heteropolymer (SAHP). The SAHP was parameterized by extracting local properties (size and shape) of blobs from the real protein (RP) trajectory.

We expect that even for a freely-jointed, self-avoiding heteropolymer (SAHP), contact probability between monomers would depend on monomer shape and separation, although a SAHP does not have tertiary structure. Inspired by the Kuhn treatment of real polymers [92], we propose that the expected intermonomer contact frequency in a SAHP can be a useful reference for detecting specific tertiary interactions (Fig 4), as long as the monomers mimic the blobs of the real protein (RP). In support of this approach, we find that within a given blob, the protein obeys Flory polymer scaling laws (S1 Text). The exponent varies across blobs (S5 Fig), capturing the intrinsic heterogeneity of the long polymer.

**Fig 5:**
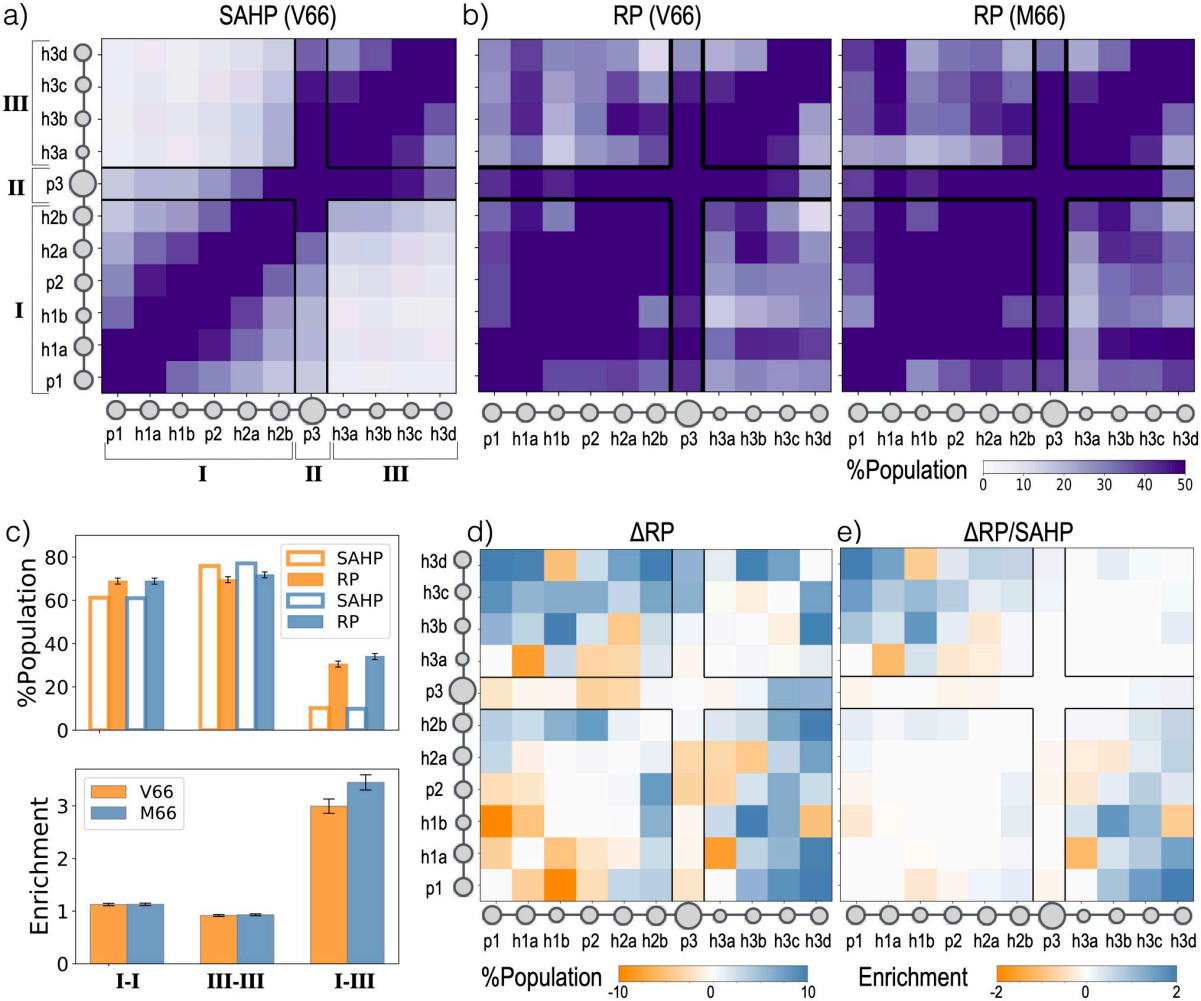
Effect of Val66Met on contacts between blobs. a) Blob-blob contact probability for the V66 self-avoiding heteropolymer (SAHP) from Monte Carlo simulations (further described in Methods). The black boxes mark the regions identified. b) Blob-blob contact probability shown in panel a for the V66 (left) and M66 (right) sequences of the real protein (RP). The x and y axes are annotated with cartoon representation of the prodomain; circles are drawn to the scale of each blob’s size. c) Population of contacts in SAHP, RP (top) and enrichment in RP contacts with respect to SAHP contacts (bottom) for each region pair. The errors represent standard errors (*n* = 1088 as described in Methods). d) Difference (M66-V66) between the contact probabilities shown in panel b. e) Differences shown in panel d with respect to SAHP; interactions more frequently found in M66 or V66 sequence are in blue and orange respectively.

The predicted contact probabilities for this freely-jointed SAHP from Monte Carlo simulations (further described in Methods) are shown in Fig 5a. In the SAHP version of the prodomain, the chain is visibly segmented by the p3 blob. As shown in S6 Fig, shifting the p3 blob within the SAHP chain shifts the visible segmentation boundary, confirming that the p3 blob defines the segmentation. Based on this expectation, we define three regions: the pre-p3 blobs are “Region I”, p3 is “Region II”, and the post-p3 blobs are “Region III”. SAHP blobs within Region I are in contact for 61% of the frames, while SAHP blobs within Region III are in contact in 76% of the frames. In comparison, the average contact probability between Regions I and III is only 10% (Fig 5c).

Fig 5b shows the probability of blob-blob contacts for both the V66 and M66 sequences of the RP, calculated analogously to those in the SAHP. The frequencies of contacts within Region I and within Region III were quantitatively consistent with the SAHP predictions. The total number of blob-blob contacts within Region I was enriched by 1.3 times the expected value for the SAHP. Within Region III, the total number was depleted by 0.9 times the expected value (Fig 5c).

In contrast, contacts between blobs on either side of the long p3 linker are more common in the RP than in the SAHP, and are also affected by the substitution at residue 66 (Fig 5c, 5d and 5e). Contacts between pre-linker Region I and post-linker Region III are about three times as common in the RP as in the SAHP, indicating specific tertiary interactions beyond those expected for a polymer undergoing a random-walk. Quantitatively, enrichment in the V66 sequence is 3.0±0.1 while enrichment in the M66 sequence is 3.4±0.1. The increased number of cross linker contacts are also consistent with the lower mean *R*_*h*_ and *R*_*g*_ for the M66 sequence.

### Effects of Val66Met on the *β*-pairing network

To test whether the changes we observed in tertiary contacts at the blob, group, or region level could be due to a change in partnering *β*-strands, we applied a clustering approach. All frames were divided into 4 clusters, representing two independent collective variables with two possible values each: either a certain contact between blobs X and Y is formed or broken, and any residue in blob X is found within a stretch of 4 sequential residues in *β* conformation. The four clusters are thus represented as (contacting,absent), (contacting,present), (distant,absent), and (distant,present).

For each cluster, we calculated *β* propensity across all residues. If the X-Y contact reflects correlated *β*-strands, we expect a peak at residues in blob Y in the (contacting,present) cluster that is significantly higher than the signal for all other clusters. If the secondary structure in Y is used for clustering instead, the reciprocal peak (at blob X) should be reproduced. Furthermore, unless there are higher-order correlations between multiple sets of *β*-strands, *β* propensity should not depend on cluster for all residues *not* in blob X or Y.

**Fig 6:**
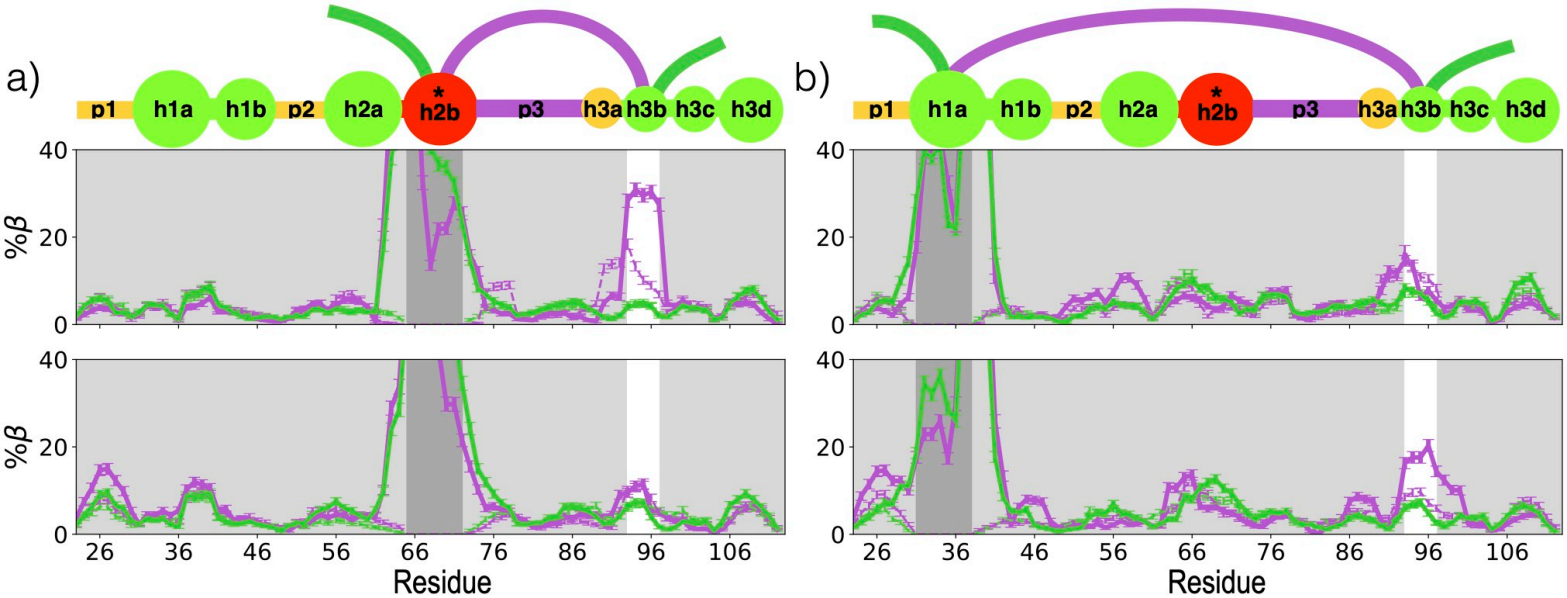
Secondary structure coupling between blobs on either side of the p3 linker. *β* propensities at each residue in the V66 sequence (top) and the M66 sequence (bottom) for four clusters. Frames were first clustered by whether the h3b-h2b (a) or h3b-h1a (b) contact was formed (purple) or broken (green), and then by whether *β* structure was present (solid) or absent (dashed) in h2b (panel a) or h1a (panel b). The dark-gray window indicates the contacting blob that is constrained to have high or vanishing values by construction of the cluster, while the white window indicates the contacting blob without constrained secondary structure. If the contact is coupled to simultaneous *β*-strand formation, the peak within the white window for the solid purple curve should be significantly higher than other curves. Errors represent standard error of a Bernoulli trial with n number of samples, where n is the product of the total number of unique replicas in a given cluster and the average number of roundtrips per replica. Panels are annotated by a blob representation of the prodomain, as in Fig 1e(i).

This clustering process on all frames was carried out for all possible X and Y blobs, provided X and Y were not in the same group and were non adjacent blobs in sequence (S7–S17 Figs). For most pairs, there was no correlating peak in *β* structure. For some pairs, a peak was present in one direction but the reciprocal peak was not present in the opposite direction. This result reflected longer *β*-strands that extended to a neighboring blob, which had the true peak. One symmetrically significant peak (indicating correlated *β* structure) involving the h3b blob was observed in each sequence. The partner blob shifted from h2b in the V66 sequence to h1a in the M66 sequence (Fig 6, S15 Fig). A second correlated pair involving the blob p1 was also observed in each sequence. The partner blob for this pair shifted from h3d in the V66 sequence to h2b in the M66 sequence (S7, S12 and S17 Figs).

Despite a loss of correlated *β*-pairing, the contact between h2b and h3b is actually more probable in the M66 sequence than in the V66 sequence (Fig 5d). As discussed in *Noteworthy residue-residue interactions stabilizing tertiary contacts*, this result reflects a significant change at the residue level. In the M66 sequence, specific interactions between M66 and side-chains of residues within h3b form the contact, rather than backbone-backbone interactions. As the h3b side-chains stabilize the contact with h2b, the backbone of h3b is then free to pair with h1a, increasing the number of favorable long-range contacts and condensing the M66 sequence overall.

### Noteworthy residue-residue interactions stabilizing tertiary contacts

**Fig 7:**
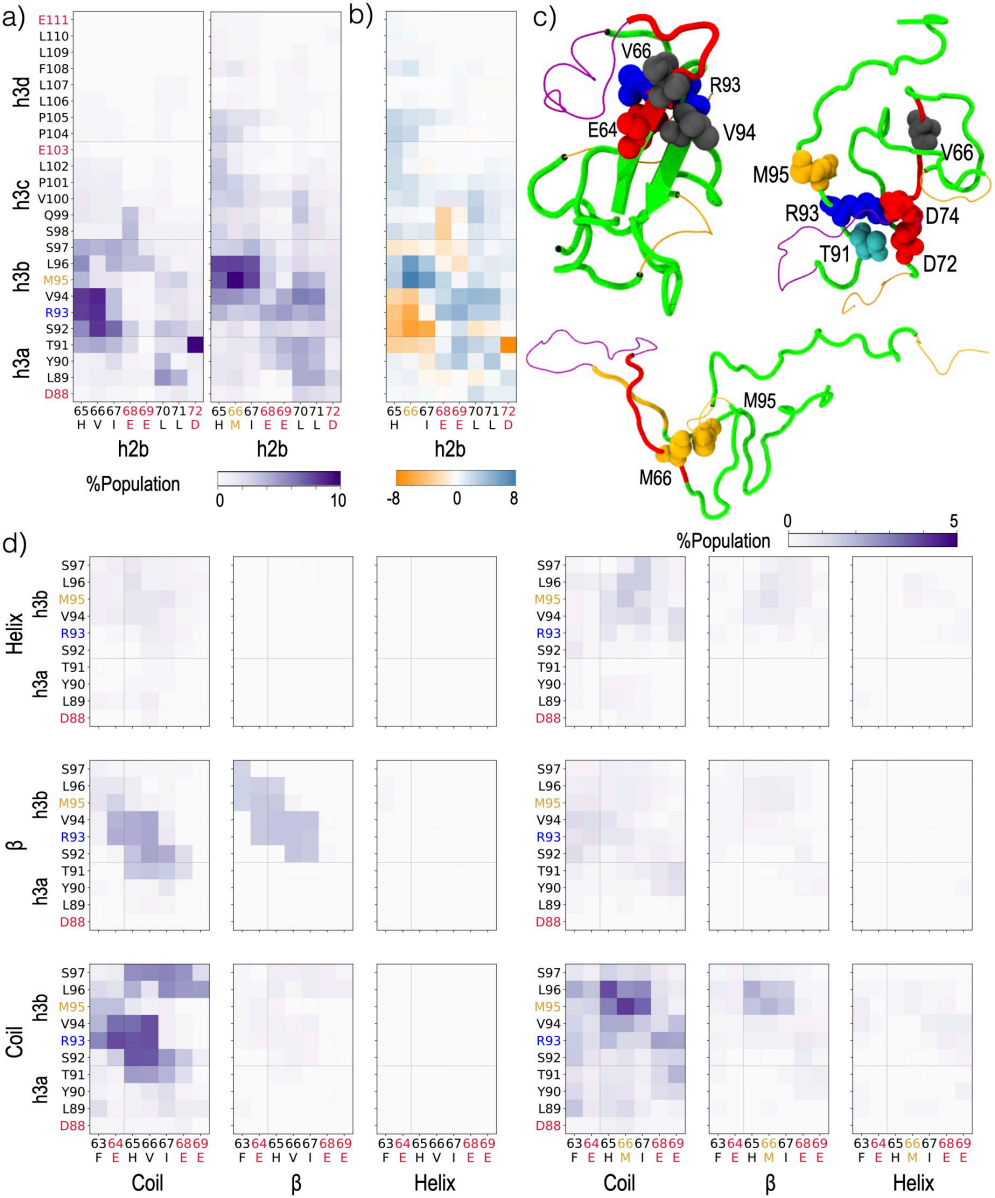
Effect of secondary structure in group h2 on which residues form the cross-boundary h2-h3 contact. a) Contact probability at each residue in h2b with each residue in h3 for V66 (left) and M66 (right) sequences. b) Difference (M66-V66) between the contact probabilities shown in panel a. c) Representative conformations of V66 sequence (top) and M66 sequence (bottom) showing preferred residue-level contacts in VDW representations. Residues are colored by residue type: blue:basic, red:acidic, cyan:polar, grey:hydrophobic except methionine, Met: yellow. The chain is colored according to the Das and Pappu diagram in Fig 1c. Tubes represent hydrophobic “h” blobs whereas lines represent non-hydrophobic linker “p” blobs. d) Contact probability between residues 63-69 and each residue in h3ab, when respective secondary structure is formed at each residue, for both the V66 (left) and M66 (right) sequences. Residue labels are colored according to residue type: blue:basic, red:acidic, grey:hydrophobic/polar and Met: yellow.

As shown previosly in *Effects of Val66Met on the *β*-pairing network*, the Val66Met substitution causes loss of correlated *β*-strands between blobs h2b and h3b, while introducing correlated *β*-strands between blobs h3b and h1a. We consider here the effects of the substitution on these contacts at residue level. As shown in the absolute residue-residue contact probability maps (Fig 7a), both sequences frequently form contacts between hydrophobic residues in blobs h2b and h3b. The residue pairs most frequently forming the contact shift from V66-V94 in the V66 sequence to M66-M95 in the M66 sequence (Fig 7b). The residue-level contact maps also show a high probability of contacts between D72 and T91 in the V66 but not M66 sequence. As illustrated in Fig 7c, these contacts (between *α* carbons) are stabilized by salt-bridges between R93 and D74, in a conformation that is incompatible with a side-chain contact between V/M66 and M95.

M95 is the only other methionine in the simulated sequence. The role of specific Met-Met interactions due to polarizable sulfur atoms is often under-appreciated, but such interactions are common in structures of folded proteins [89]. Using ab initio calculations, Gómez-Tamayo et al. [91] predicted that Met-Met interactions are stronger than Met-aromatic or aromatic-aromatic interactions, due to the polarizability of sulfur. Although the fixed-charge force-field we are using (a99sb*-ildn-q) cannot explicitly capture polarizability, Gómez-Tamayo et al. demonstrate that this force-field preserves rankings of strong side-chain interactions involving methionine. In these simulations, the M66-M95 contact was about five times as common (10% of frames) as the analogous V66-M95 contact (2% of frames) (Fig 7a and 7b). Methionine-aromatic interactions also contribute to the increased number of Region I-III contacts: M66, but not V66, forms a frequent contact with F108 in blob h3d, which is also consistent with the favorable interactions between Met-Phe residues [88–90] (Fig 7a and 7b).

To determine which residue contacts between h2b and h3ab couple the secondary structure within the two blobs, we decomposed the residue-level contact maps into nine clusters. Each cluster was specified by two collective variables with three possible values each: secondary structure (helix, *β*, or coil) around residue 66 and secondary structure (helix, *β*, or coil) in h3ab (Fig 7d). The *β*-pairing at h2b-h3ab is stabilized via a combination of backbone hydrogen bonds between V66 and S92, salt-bridge between E64 and R93, and hydrophobic interactions between V66 and V94. The V66-M95 contact was only formed frequently within the (h2b—coil, h3ab—helix) cluster, and since this cluster was a very small part of the overall population, the contact overall was rare as well (Fig 7d). This cluster was more common in the M66 sequence, and contributes to the non-local increase in helicity around residue 95 (Fig 3b).

### Summary and conclusion

We have carried out over 250 *μ*s of fully-atomistic explicit solvent MD simulation of the 91 residue BDNF prodomain, with and without the disease-associated Val66Met mutation. These long simulations successfully reproduced the experimentally observed secondary chemical shifts and hydrodynamic radius. The simulations also correctly reproduced the location of both local and non-local secondary changes due to the Val66Met mutation in the BDNF prodomain.

We find that the highly disordered 91 residue prodomain, which as a whole falls in the Janus sequence region of the Das and Pappu phase diagram [21], can be meaningfully divided into 11 blobs based on sequence hydrophobicity alone. Among 8 hydrophobic blobs, we identified 2 blobs in the disordered region: the strong polyelectrolyte blob h2b (which contains Val66Met), and the Janus blob h3a. These are connected via the highly disordered long linker p3. The groups containing these unique blobs have biological significance as well: The sequence h2-p3-h3 is essential for intracellular trafficking of precursor BDNF [93].

We used the protein sequence to systematically design a tractable approach for coarse-graining analysis, by reducing the initial number of potential contacts from over 4000 to 55, while increasing the number of observations for each contact. Furthermore, it allowed us to isolate the most sensitive regions of the protein for examination at the residue level. This method, simply based on sequence hydrophobicity, may be a generally useful informatics strategy to suggest functionally significant regions in long disordered proteins. Our conclusions further suggest an important role for disorder heterogeneity within disordered proteins.

We were able to identify mechanisms through which a charge-neutral mutation can affect the residual secondary structure and tertiary contacts of a disordered protein. We further identified how these effects can be propagated to non-local residual secondary structure. Within its local blob h2b, the Val66Met mutation affects local contact preference due to local sequence effects (preferred Met-Phe contacts) and the reduced entropic cost of helix formation for the methionine sidechain.

The long, disordered, exposed Region II linker segregates the blob-level contact probability map: blobs within Region I or Region III have a high probability of contact, while Region I-III contacts are far less probable. We consistently observed this segregation in both simple self-avoiding hetropolymer simulations with beads mimicking identified blobs, and actual prodomain simulations. Val66Met increases the frequency of Region I-III contacts. We find here that the dominant mechanism involves replacing *β*-strand coupling between group h2 of Region I and group h3 of Region III with favorable Met/Met side-chain interactions between the same groups. The group h3 backbone is then exposed for interactions with the backbone of group h1, also of Region I. The non-local increase in helicity in group h3 may reflect stabilization of non-*β* structure by the Met-Met interactions.

Met/Met interactions have been shown to stabilize tertiary contacts in folded proteins and membrane proteins, but their role has not been investigated in disordered proteins. In general, our study supports previous observations [91, 94] that methionine plays a distinct role from true aliphatic residues in determining protein structure, and highlights the importance of mimicking its unique properties within fixed-charge force-fields.

Anastasia et al. [63] observed differential kinetics for interactions between the BDNF prodomain and SorCS2, and also observed that the SNP-containing blob h2b (H65 to L71) only interacts with SorCS2 in the M66 sequence. The increased interactions between M66 and SorCS2 could be attributed to increased helical propensity at that residue and/or specific Met-Met contacts. In the first mechanism, helix formation in the SNP blob segregates acidic and hydrophobic residues on opposite sides of the helix. It is possible that this preformed structure will stabilize binding. The second mechanism is suggested by the specific Met-Met interactions we observed in the isolated prodomain, as well as the high number of exposed methionines on the SorCS2 surface. It is also possible both mechanisms could contribute to stabilizing the complex, although this would require a more specific protein-protein interface.

## Methods

### System setup

To account for differences in starting coil conformation, we included six unique structures to represent residues 23-113 of BDNF prodomain. These structures were built using I-Tasser [95–97], Rosetta [98] or Modeller [99], and were simulated in a water box at 600K for 50 ns at a constant volume. From the six resulting trajectories, 64 structures with correct proline isomers were selected (based on at least 2ps time interval); in total, our study included 64 unique prodomain structures. All structures were cooled to 300K for 1ns, while prolines were restrained in trans-conformation. Each V66 replica was placed in a dodecahedron water box with approximately 30,500 Tip4p-D [74] water molecules and a 0.15M salt concentration (NaCl) for a total system size of approximately 124,000 atoms. The same volume for each replica was ensured by fixing the simulation box of each replica to the average box size (11 nm).

### MD simulations

For the simulations we use the a99sb*-ildn-q force-field [78, 79] and the GROMACS 5.1.2 simulation package [100, 101], with a time step of 2 fs. Long-range electrostatics are calculated using the particle mesh Ewald (PME) method [102], with a 1 nm cutoff and a 0.12 nm grid spacing. Periodic boundary conditions are also used to reduce system size effects. The system was simulated using T-REMD [103] with an exchange frequency of 1ps for 2 *μ*s, giving a total simulation time of 128 *μ*s with NVT ensemble for each system. 64 replicas are used with temperatures ranging from 300-385K, with exponential spacing. A different random seed was used for the Langevin dynamics of each replica. The average exchange acceptance probability ranged between 0.19-0.23.

**Fig 8:**
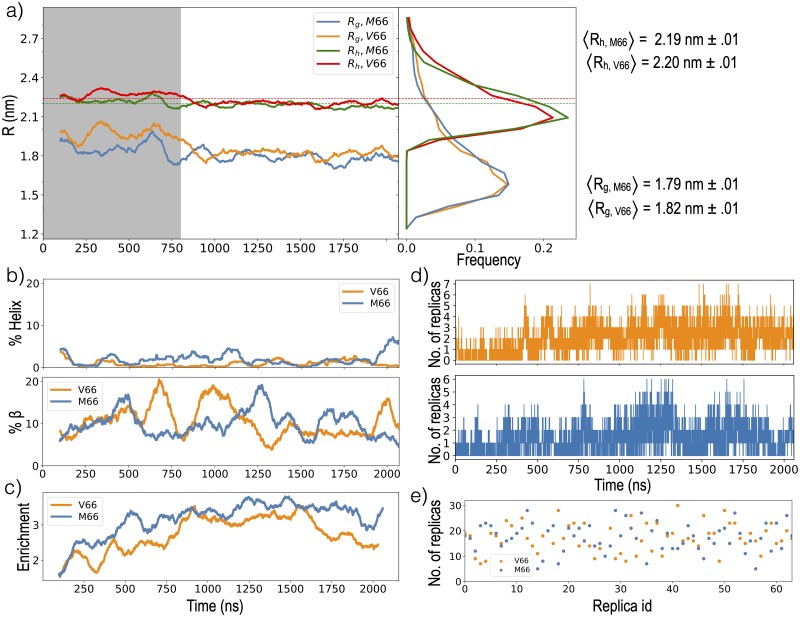
Simulation convergence. a) Trajectory (left) and distribution (right) of *R*_*g*_ and *R*_*h*_ for the 300K replicas. The shaded region represents the equilibration period discarded from the distribution and from all analysis presented in *Results and Discussion*. Experimental values of *R*_*h*_ from NMR diffusion [63] are 2.24±0.1 nm for the V66 sequence and 2.20±0.1 nm for the M66 sequence, and are indicated by dashed lines. b) Trajectory of helix (top) and *β* (bottom) propensity at residue 66 for the 300K replicas of both sequences. c) Trajectory of enrichment of total Region I-Region III contacts relative to SAHP in the 300K replica. The trajectory shows averages over a 100 ns moving window in panel a, b and c. d) Number of replicas forming the V66-V94 contact (top) and the M66-M95 contact (bottom) vs the simulation time. e) The number of round trips completed by each replica over the 1.2 *μ*s production period.

The minimum separation between the molecule and its image was less than 2 nm for less than 1% of the frames for both sequences and these frames were discarded from all analysis. Time-series of the relative measurements were generated every 100 ps. For both V66 and M66 sequences, initial 51.2 *μ*s (800 ns × 64) trajectories were discarded for equilibration purposes, determined by plateauing of *R*_*g*_ (Fig 8a). Over the course of remaining 76.8 *μ*s (1.2 *μ*s × 64) simulations, each replica completes a minimum of 5 roundtrips and an average of 17 roundtrips for each sequence (Fig 8e). Simulation convergence was monitored using several metrics (Fig 8).

Time-series of the *R*_*g*_ and end to end distance (*R*_*etoe*_) were calculated using respectively the g_gyrate and g_polystat utilities of Gromacs. We took *R*_*etoe*_ as the distance between N-termini and C-termini N and O atoms respectively. Statistical uncertainties are provided for *R*_*etoe*_ and *R*_*g*_ as the standard error in the mean, where *n* = 1088 is the product of the total number of replicas simulated (64) and the average number of roundtrips per replica (17).

### Blob identification

〈*H*〉 at each residue is defined as the average Kyte-Dolittle [65] score with a window size of 3 residues, scaled to fit between 0 and 1. Any stretch of four or more residues with 〈*H*〉 > 0.37 is classified as a hydrophobic or “h” blob and any remaining stretch of four or more residues is classified as a non-hydrophobic linker or “p” blob. Multiple consecutive hydrophobic blobs without a “p” blob separating them are classified as a single group.

### Secondary chemical shifts

Prior to the present study, Anastasia et al. [63] measured chemical shifts for the BDNF prodomain (residues 21-113) using NMR, and then used backbone NMR secondary chemical shifts to predict secondary structure via TALOS+ [104] and SSP [105]. For comparison with simulation data, we reinterpreted the chemical shifts directly from [63], deposited at Biological Magnetic Resonance Bank (BMRB). C_*α*_ secondary chemical shifts are calculated as follows: Δ*δC*_*α*,*MD*_ = (*δC*_*α*,*MD*_ − *δC*_*α*,*RC*(300*K*)_) and Δ*δC*_*α*,*NMR*_ = (*δC*_*α*,*NMR*_ − *δC*_*α*,*RC*(280*K*)_), where *δC*_*α*,*MD*_, *δC*_*α*,*NMR*_ and *δC*_*α*,*RC*_ are predicted C_*α*_ chemical shifts from MD simulation, NMR experiments and random coil respectively. *δ*C_*α*_ were calculated from MD simulated conformations using SPARTA+ [83]. NMR experiments values were obtained from the data deposited at BMRB by Anastasia et al [63]. Random coil *δ*C_*α*_ for the 91 residue BDNF prodomain were obtained using POTENCI [106] at pH 7, with a 0.15 M ion concentration, at 280K and 300K for NMR and MD respectively. Error at each residue is calculated as the standard error in the mean, where *n* = 1088 is the product of the total number of replicas simulated (64) and the average number of roundtrips per replica (17). C_*β*_ secondary chemical shifts were calculated analogously.

### Hydrodynamic radius calculation

The values for the Hydropro [86] parameters were: atomic level model with shell-method calculation, a = 0.29 nm, 6 minibead iterations, and *σ* = 0.1 to 0.2 nm. The temperature was taken to be 300 K, the solvent viscosity was 0.01 Poise, the solvent density was 1.0 gcm^−3^, the partial specific volume of the peptide 0.7313 cm^3^g^−1^ (V66 sequence) or 0.7304 cm^3^g^−1^ (M66 sequence), and molecular weight of the peptide was equal to 10044 Da (V66 sequence) or 10076 Da (M66 sequence). The resultant translational diffusion constants were then used for calculating *R*_*h*_ using the Stokes-Einstein equation. Error is calculated as the standard error in the mean, where *n* = 1088 is the product of the total number of replicas simulated (64) and the average number of roundtrips per replica (17).

### Secondary structure calculation

Helix propensity or *β* propensity is expressed as the probability of a given residue being part of a sequence of four consecutive residues whose dihedral angles place them in the helical region or *β* region of the Ramachandran space. The helical region is defined as -100° <*ϕ* <-30° and -120° ≤ *ψ* ≤ 50° [42, 107, 108]. The *β* region is defined as *ϕ* <-80° and *ψ* > 50° or *ψ* < 120°. The error bars are the standard error of a Bernoulli trial with n number of samples, where n is the product of the total number of unique replicas in a cluster and the average number of roundtrips per replica. The length of secondary structure (SS-map) [109] were calculated with the above defined helical and *β* region.

### Blob-level contact maps

**Fig 9:**
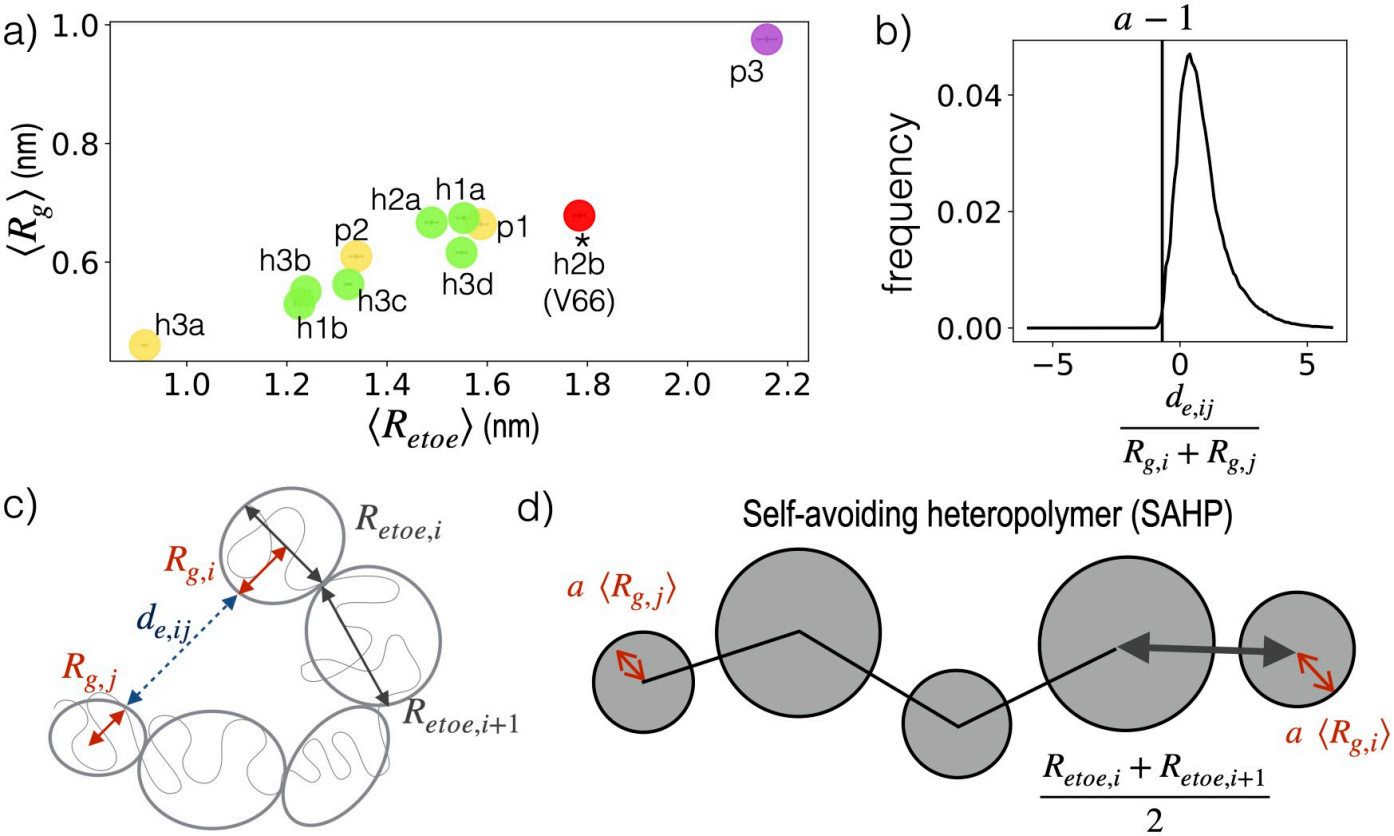
Parameterization of self-avoiding heteropolymer. a) 〈*R*_*g*_〉 vs 〈*R*_*etoe*_〉 for each blob of V66 sequence. Blobs are colored according to the Das and Pappu diagram in Fig 1c. Statistical error was smaller than the circles used for the representation of each blob. b) The distribution of normalized excess distances across all blob-pairs in the V66 RP, where |*i* − *j*| > 1. c) Relationship between the radius of gyration *R*_*g*,*i*_, end to end distance *R*_*etoe*,*j*_, and excess distance *d*_*ij*_, calculated for each blob or blob pair using a RP trajectory. d) The SAHP is a chain with each monomer representing a blob of the RP and modeled as a hard sphere. Each monomer *i* has radius *aR*_*g*,*i*_ and is separated from monomer *i* + 1 by bond length (*R*_*etoe*,*i*_ + *R*_*etoe*,*i*+1_)/2. Bond lengths are constrained and bond angles can rotate freely.

As illustrated in Fig 9c, the excess distance between any two blobs *i* and *j* is defined as

$$\begin{array}{c} d_{e,ij}=|{\vec{r}}_{i}-{\vec{r}}_{j}|-\left(R_{g,i}+R_{g,j}\right) \end{array}$$ (1)

where ${\vec{r}}_{i}$ is the position vector of a blob *i* defined as the mean of its N-terminal N atom and the C-terminal O atom coordinates, calculated using g_traj utility of Gromacs. Two blobs *i* and *j* are in contact if the excess distance (*d*_e,ij_) between the two is less than 0.55 nm. At residue level, two residues are in contact if the distance between C_*α*_ atoms of the two residues is 0.8 nm or less. Presented statistical uncertainties are the standard error in the mean, with n is the product of the total number of replicas forming the given contact and the average number of roundtrips per replica.

### Self-avoiding heteropolymer simulation

The BDNF prodomain was approximated as a freely-jointed self-excluding heteropolymer with 11 monomers, each mimicking one of the blobs identified in Fig 1b. As illustrated in Fig 9d), the separation between monomers *i* and *i* + 1 (analogous to the Kuhn length for a homopolymer [92]) was constrained to be half the end to end distance for each of the analogous blobs:

$$\begin{array}{c} |{\vec{r}}_{i-1}-{\vec{r}}_{i}|=\frac{\left\langle R_{etoe,i-1}\right\rangle +\left\langle R_{etoe,i}\right\rangle }{2} \end{array}$$ (2)

where 〈*R*_*etoe*,*i*_〉 was determined from the coordinates of blob *i* residues in the MD simulations, shown in Fig 9a.

Two monomers *i* and *j* are considered to be overlapping if

$$\begin{array}{c} \frac{|{\vec{r}}_{i}-{\vec{r}}_{j}|}{\left\langle R_{g,i}\right\rangle +\left\langle R_{g,j}\right\rangle }=\frac{d_{e,ij}}{\left\langle R_{g,i}\right\rangle +\left\langle R_{g,j}\right\rangle }+1<a \end{array}$$ (3)

where 〈*R*_*g*,*i*_〉 was determined from the coordinates of residues in blob *i* in the MD simulations (Fig 9a), and *a* is a constant. In the MD simulations of the real protein, we observed that $\frac{d_{e,ij}}{\langle R_{g,i}\rangle +\langle R_{g,j}\rangle }\geq -0.7$ for almost all frames (Fig 9b), and thus we set *a* = 0.3.

The random walk was carried out using a simple Metropolis Monte Carlo, with the following move set: 1) a random bead *i* > 0 was selected, 2) a random displacement vector $\vec{\delta r}$ of magnitude 0.5 nm was generated in three cartesian dimensions, 3) $\vec{\delta r}$ was scaled so that $|{\vec{r}}_{i-1}-\left({\vec{r}}_{i}+\vec{\delta r}\right)|=\left(\langle R_{etoe,i-1}\rangle +\langle R_{etoe,i}\rangle \right)/2$, satisfying Eq 2, 4) the translation ${\vec{r}}_{j}\to \vec{r_{j}}+\delta_{r}$ was applied for all *j* ≥ *i*.

Any trial move that caused an overlap according to Eq 3 was rejected, while all others were accepted. The Monte Carlo simulation was run for 5,000,000 steps (500,000 steps per moveable bead); additional steps did not change the outcome in Fig 5a.

## Supporting information

S1 Table Summary of force-field comparison simulations. (PDF)

S1 Text Heterogeneous behavior of individual blobs. (PDF)

S1 Fig Force-field comparison. We ran T-REMD simulations of a 30 residue fragment of the V66 prodomain with several commonly used force-field and water model combinations. (a) Comparison of Δ*δ*C_*α*_ at 280K from MD ensembles for a99sb*-ildn-q [78, 79] with Tip4p-D [74], c36m [80], a99sbws [76, 78], a03sbws [75, 76], a99sb-ildn with Tip3p [81], calculated using SPARTA+ [83] and NMR from Ref. [63]. (b) *R*_*g*_ vs the simulation time, using a 100 ns moving window on left and *R*_*g*_ distribution for each force-field on right. Tip3p and a03sbws generates most collapsed and expanded *R*_*g*_ distribution respectively. The equilibration time and 〈*R*_*g*_〉 is shown with vertical and horizontal dashed lines for each force-field. The *R*_*g*_ distribution and its mean does not include the simulation equilibration time. The 〈*R*_*g*_〉 values are also reported in S1 Table.
(TIFF)

S2 Fig Effects of temperature and Val66Met mutation on helix propensity around residue 66. The frequency of formation of a helix of a given length containing residue 66 in V66 (top) and M66 (bottom) sequences in the temperature range of 300K to 385 K. With the increase in temperature the color transitions from cooler (blue) to hotter (red). It is entropically unfavorable for V66 and its neighboring residue to be simultaneously in the helical region of the Ramachandran map, as indicated by the decreasing helical propensity with increasing temperature. For longer helices, the trend will depend more on the additional side-chains in the helix, and the trend with temperature is reversed, but it remains weaker than the analogous trend for the M66 sequence. Errors represent the standard error of a Bernoulli trial with n number of samples, where n is the product of the total number unique replicas forming the helix of given length at residue 66 at a given temperature and the average number of roundtrips per replica, 17.
(TIFF)

S3 Fig Residue level contacts for the entire prodomain. a) Contact probability between every residue pair for V66 (left), M66 (middle) sequences and the difference between the two (right). Two residue pairs are considered to be in contact if the *C*_*α*_-*C*_*α*_ distance between the two residues is less than or equal to 0.8 nm. Panels are annotated by a blob representation of the prodomain, as in Fig 1e(i); vertical and horizontal grey lines in each panel represent the blob boundaries. b) A linear network of transient tertiary contacts shown in panel a. The contact networks were build using Cytoscape [110] with a linear representation of residues. Each protein residue comprises a node in the network, with interactions between residues represented as edges. The strength of individual interactions can be interpreted by the thickness of the edge line on the network diagram. If the separation between residues forming the contact is more than 20, its edge is drawn above the node; otherwise, the edge is drawn at the bottom of the node. To focus on significant interactions, interactions showing more than 6% persistence were considered in the network visualization. The x axis is annotated with blob representation of the prodomain, as in Fig 1e(i).
(TIFF)

S4 Fig Residue level contacts for the entire prodomain including backbone-backbone, sidechain-sidechain, salt-bridge and hydrophobic contacts. Contact probability between every residue pair for V66 (left) and M66 (middle) sequences and the difference between the two (right). Two residue pairs are in contact if the distance between backbone-backbone atoms between the two residues are 0.4 nm or less (1st row), if the distance between non hydrogen sidechain-sidechain atoms between the two residues are 0.4 nm or less (2nd row), if the distance between non hydrogen sidechain-sidechain atoms between the two hydrophobic residues are 0.4 nm or less (3rd row), if the two residue pairs are forming a salt-bridge with the distance between the donor and acceptor atoms < 0.32 nm (4th row). Panels are annotated by a blob representation of the prodomain, as in Fig 1e(i); vertical and horizontal grey lines in each panel represent the blob boundaries.
(TIFF)

S5 Fig Polymer scaling behavior for each identified blob and entire prodomain. a) Mean distances between any residues *i* and *j* at 300K, for the entire V66 and M66 prodomains as well as each blob in the V66 (left) and M66 (right) sequences. Theoretical polymer scaling limits are represented by the curves 〈*R*_|*i*−*j*|_〉 = *A*|*i* − *j*|^*ν*^ where *A* = 0.59 nm and *ν* is the Flory exponent. For good, theta, and bad solvent, *ν* = 3/5, 1/2, 1/3 respectively. b) Values of *ν* resulting from fits to each blob for V66 (left) and M66 (right) sequences. The x axis is annotated with a blob representation of the prodomain where blobs are colored according to the Das and Pappu diagram [21] in Fig 1c.
(TIFF)

S6 Fig Effect of perturbing monomer properties on freely-jointed, self-avoiding heteropolymer. Contact probability maps from SAHP calculations, analogous to those in Fig 5a of the main text. The x and y axes are annotated with cartoon representation of the prodomain; circles are drawn to the scale of each blob’s size. Here the SAHP model is varied systematically by swapping the p3 blob with every other blob in the chain. As the p3 blob is shifted along the chain, p3 and p1 consistently bound a white “forbidden” region that has little interaction with the rest of the protein.
(TIFF)

S7 Fig *β*-pairing between blob p1 and each remaining blob, excluding adjacent or intra group pairs in the V66 (left) and M66 (right) sequences. Frames were first clustered by whether the X-Y contact was formed (purple) or broken (green), and then by whether *β* structure was present in X (solid) or absent (dashed). The dark-gray window indicates the contacting blob that is constrained to have high or vanishing values by construction of the cluster, while the white window indicates the contacting blob without constrained secondary structure. If the contact is coupled to simultaneous *β*-strand formation, the peak within the white window for the solid purple curve should be significantly higher than other curves. Errors represent standard error of a Bernoulli trial with n number of samples, where n is the product of total number of unique replicas in a given cluster and average number of roundtrips per replica (17). X represents p1 and Y represents other blobs identified in the sequence and is annotated on the left for each panel.
(TIFF)

S8 Fig *β*-pairing between blob h1a and each remaining blob, excluding adjacent or intra group pairs in the V66 (left) and M66 (right) sequences. Same as S7 Fig, but for h1a blob.
(TIFF)

S9 Fig *β*-pairing between blob h1b and each remaining blob, excluding adjacent or intra group pairs in the V66 (left) and M66 (right) sequences. Same as S7 Fig, but for h1b blob.
(TIFF)

S10 Fig *β*-pairing between blob p2 and each remaining blob, excluding adjacent or intra group pairs in the V66 (left) and M66 (right) sequences. Same as S7 Fig, but for p2 blob.
(TIFF)

S11 Fig *β*-pairing between blob h2a and each remaining blob, excluding adjacent or intra group pairs in the V66 (left) and M66 (right) sequences. Same as S7 Fig, but for h2a blob.
(TIFF)

S12 Fig *β*-pairing between blob h2b and each remaining blob, excluding adjacent or intra group pairs in the V66 (left) and M66 (right) sequences. Same as S7 Fig, but for h2b blob.
(TIFF)

S13 Fig *β*-pairing between blob p3 and and each remaining blob, excluding adjacent or intra group pairs in the V66 (left) and M66 (right) sequences. Same as S7 Fig, but for p3 blob.
(TIFF)

S14 Fig *β*-pairing between blob h3a and each remaining blob, excluding adjacent or intra group pairs in the V66 (left) and M66 (right) sequences. Same as S7 Fig, but for h3a blob.
(TIFF)

S15 Fig *β*-pairing between blob h3b and each remaining blob, excluding adjacent or intra group pairs in the V66 (left) and M66 (right) sequences. Same as S7 Fig, but for h3b blob.
(TIFF)

S16 Fig *β*-pairing between blob h3c and each remaining blob, excluding adjacent or intra group pairs in the V66 (left) and M66 (right) sequences. Same as S7 Fig, but for h3c blob.
(TIFF)

S17 Fig *β*-pairing between blob h3d and each remaining blob, excluding adjacent or intra group pairs in the V66 (left) and M66 (right) sequences. Same as S7 Fig, but for h3d blob.
(TIFF)
