# Supplementary material for: Sequence specificity despite intrinsic disorder: How a disease-associated Val/Met polymorphism rearranges tertiary interactions in a long disordered protein
Source: PLoS Comput Biol. 2019 Oct 18;15(10):e1007390. doi: 10.1371/journal.pcbi.1007390 (PMC6821141; doi:10.1371/journal.pcbi.1007390)
Supplement: S1 Table — (PDF) [file pcbi.1007390.s001.pdf]

Table S1: **Summary of force-field comparison simulations.**

Force-field	RMSD$_a$ $\Delta\delta C_\alpha$	$\langle R_g \rangle^b$	Equilibration$_c$ length	No. of $_d$ replicas	Temperature range^e	Simulation$_f$ length
aff03sbws (Tip4p/2005)	0.855	1.347 ±0.007	400 ns	36	280-360K	700 ns
a99sb*-ildn-q (Tip4p-D)	0.355	1.270 ±0.007	200 ns	36	280-360K	500 ns
a99sbws (Tip4p/2005)	0.425	1.277 ±0.007	200 ns	36	280-360K	500 ns
c36m (Tip3p)	0.350	1.306 ±0.007	200 ns	30	280-360K	500 ns
a99sb-ildn (Tip3p)	0.617	0.922 ±0.003	200 ns	32	280-420K	500 ns

[a] Root-mean-squared deviation (RMSD) represents the deviation between the NMR and MD $\Delta\delta C_\alpha$.

[b] Statistical uncertainties are provided for $\langle R_g \rangle$ as the standard error in the mean, where n is the product of the total number of replicas simulated and the average number of roundtrips per replica.

[c] Simulation period discarded for equilibration for each replica.

[d] Total number of replicas simulated using T-REMD.

[e] Temperature range for T-REMD.

[f] Total simulation length for each replica.
