# Supplementary material for: Sequence specificity despite intrinsic disorder: How a disease-associated Val/Met polymorphism rearranges tertiary interactions in a long disordered protein
Source: PLoS Comput Biol. 2019 Oct 18;15(10):e1007390. doi: 10.1371/journal.pcbi.1007390 (PMC6821141; doi:10.1371/journal.pcbi.1007390)
Supplement: S1 Text — (PDF) [file pcbi.1007390.s002.pdf]

Heterogeneous behavior of individual blobs

We also calculated the polymer properties of each blob. Disordered proteins can be well-described by Flory scaling theory $\langle R_{|i-j|} \rangle = A|i-j|^{\nu}$, where $\langle R_{|i-j|} \rangle$ is the ensemble-averaged internal distance, |i-j| is residue separation along the chain, and ν is the Flory scaling coefficient [1]. Larger values of ν correspond to swollen coils, while smaller values correspond to compact globules [2]. In particular, when ν=0.6 ("good solvent") the protein maximizes its interaction with solvent, and for ν=0.33 ("poor solvent"), the protein maximizes self-interactions. The special intermediate case of ν=0.5 is called a "theta solvent" [1]. Most IDPs that obey this scaling behavior have ν>0.5 [3, 2, 4, 5].

As shown in S5 Fig the prodomain as a whole is not well fit by a single power law: for separations of 15 or fewer residues the prodomain falls in the "theta solvent" regime, while for separations of 20 or more residues it falls in the "poor solvent" regime. Each identified individual blob does obey a power law, and we calculated A and ν for each blob as if it was isolated from rest of the protein (S5 Fig). The highest observed value of ν was in blob h2b and h3c. This is in agreement with strong polyelectrolyte nature of h2b and high content of Proline residue (20%) in h3c.

Method: We calculated the average distance between the first atom (N) and last atom (O) for all residue pairs of a given sequence as a function of sequence separation $|i-j|$ using g_traj. Errors before fitting were calculated as the standard error in the mean, where $n = 1088$ is the product of the total number of replicas simulated (64) and the average number of roundtrips per replica (17). ν was calculated by linear fit of $\ln(\langle R_{|i-j|} \rangle)$ vs $\ln(|i-j|)$ weighted by each point's pre fit error with fixed A of 0.59 nm. To exclude the short-range backbone rigidity, distances with $|i-j| < 3$ were not fit.

References

[1] Flory PJ. The Configuration of Real Polymer Chains. J Chem Phys. 1949;17(3):303–310. doi:10.1063/1.1747243.

[2] Das RK, Pappu RV. Conformations of intrinsically disordered proteins are influenced by linear sequence distributions of oppositely charged residues. Proc Natl Acad Sci. 2013;110(33):13392–13397. doi:10.1073/pnas.1304749110.

[3] Hofmann H, Soranno A, Borgia A, Gast K, Nettels D, Schuler B. Polymer scaling laws of unfolded and intrinsically disordered proteins quantified with single-molecule spectroscopy. Proc Natl Acad Sci. 2012;109(40):16155–16160. doi:10.1073/pnas.1207719109.

[4] Zerze GH, Best RB, Mittal J. Sequence- and Temperature-Dependent Properties of Unfolded and Disordered Proteins from Atomistic Simulations. J Phys Chem B. 2015;119(46):14622–14630. doi:10.1021/acs.jpcb.5b08619.

[5] Meng F, Bellaiche MMJ, Kim JY, Zerze GH, Best RB, Chung HS. Highly Disordered Amyloid-β Monomer Probed by Single-Molecule FRET and MD Simulation. Biophys J. 2018;114(4):870–884. doi:10.1016/j.bpj.2017.12.025.
