# Supplementary figures and images for: Sequence specificity despite intrinsic disorder: How a disease-associated Val/Met polymorphism rearranges tertiary interactions in a long disordered protein
Source: PLoS Comput Biol. 2019 Oct 18;15(10):e1007390. doi: 10.1371/journal.pcbi.1007390 (PMC6821141; doi:10.1371/journal.pcbi.1007390)

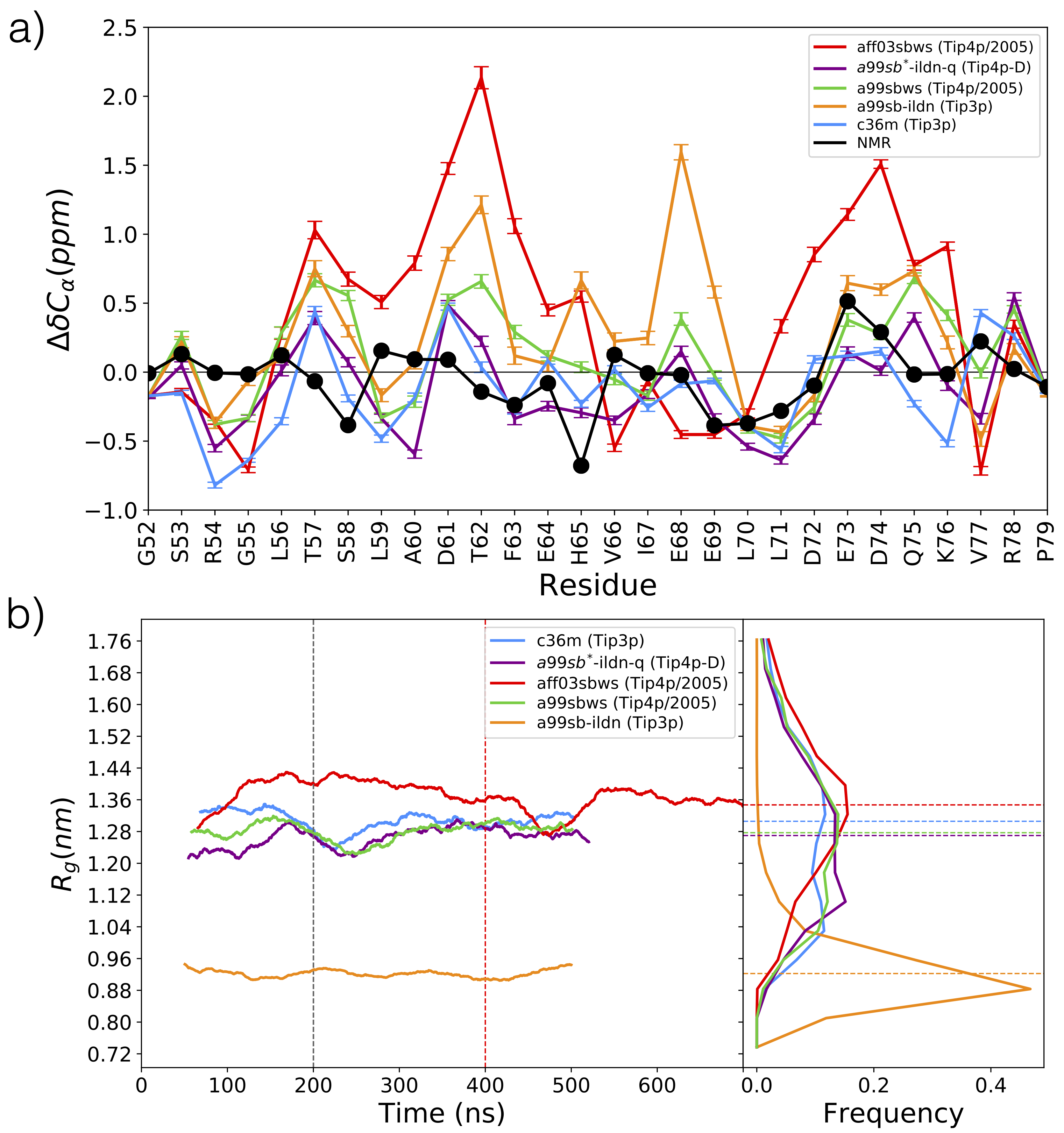

Supplement: S1 Fig — We ran T-REMD simulations of a 30 residue fragment of the V66 prodomain with several commonly used force-field and water model combinations. (a) Comparison of ΔδCα at 280K from MD ensembles for a99sb*-ildn-q [78, 79] with Tip4p-D [74], c36m [80], a99sbws [76, 78], a03sbws [75, 76], a99sb-ildn with Tip3p [81], calculated using SPARTA+ [83] and NMR from Ref. [63]. (b) Rg vs the simulation time, using a 100 ns moving window on left and Rg distribution for each force-field on right. Tip3p and a03sbws generates most collapsed and expanded Rg distribution respectively. The equilibration time and 〈Rg〉 is shown with vertical and horizontal dashed lines for each force-field. The Rg distribution and its mean does not include the simulation equilibration time. The 〈Rg〉 values are also reported in S1 Table. (TIFF) [file pcbi.1007390.s003.tiff]

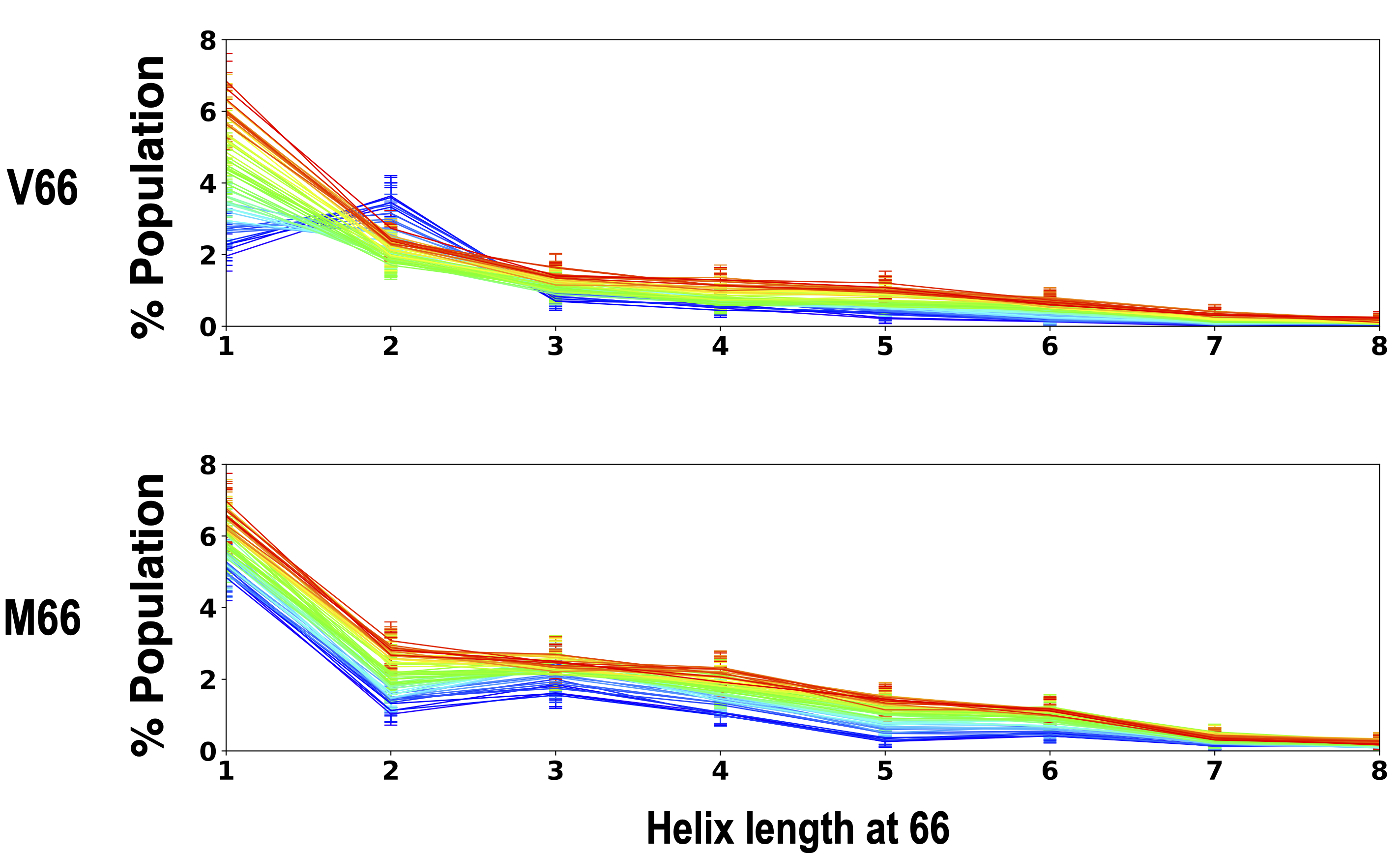

Supplement: S2 Fig — The frequency of formation of a helix of a given length containing residue 66 in V66 (top) and M66 (bottom) sequences in the temperature range of 300K to 385 K. With the increase in temperature the color transitions from cooler (blue) to hotter (red). It is entropically unfavorable for V66 and its neighboring residue to be simultaneously in the helical region of the Ramachandran map, as indicated by the decreasing helical propensity with increasing temperature. For longer helices, the trend will depend more on the additional side-chains in the helix, and the trend with temperature is reversed, but it remains weaker than the analogous trend for the M66 sequence. Errors represent the standard error of a Bernoulli trial with n number of samples, where n is the product of the total number unique replicas forming the helix of given length at residue 66 at a given temperature and the average number of roundtrips per replica, 17. (TIFF) [file pcbi.1007390.s004.tiff]

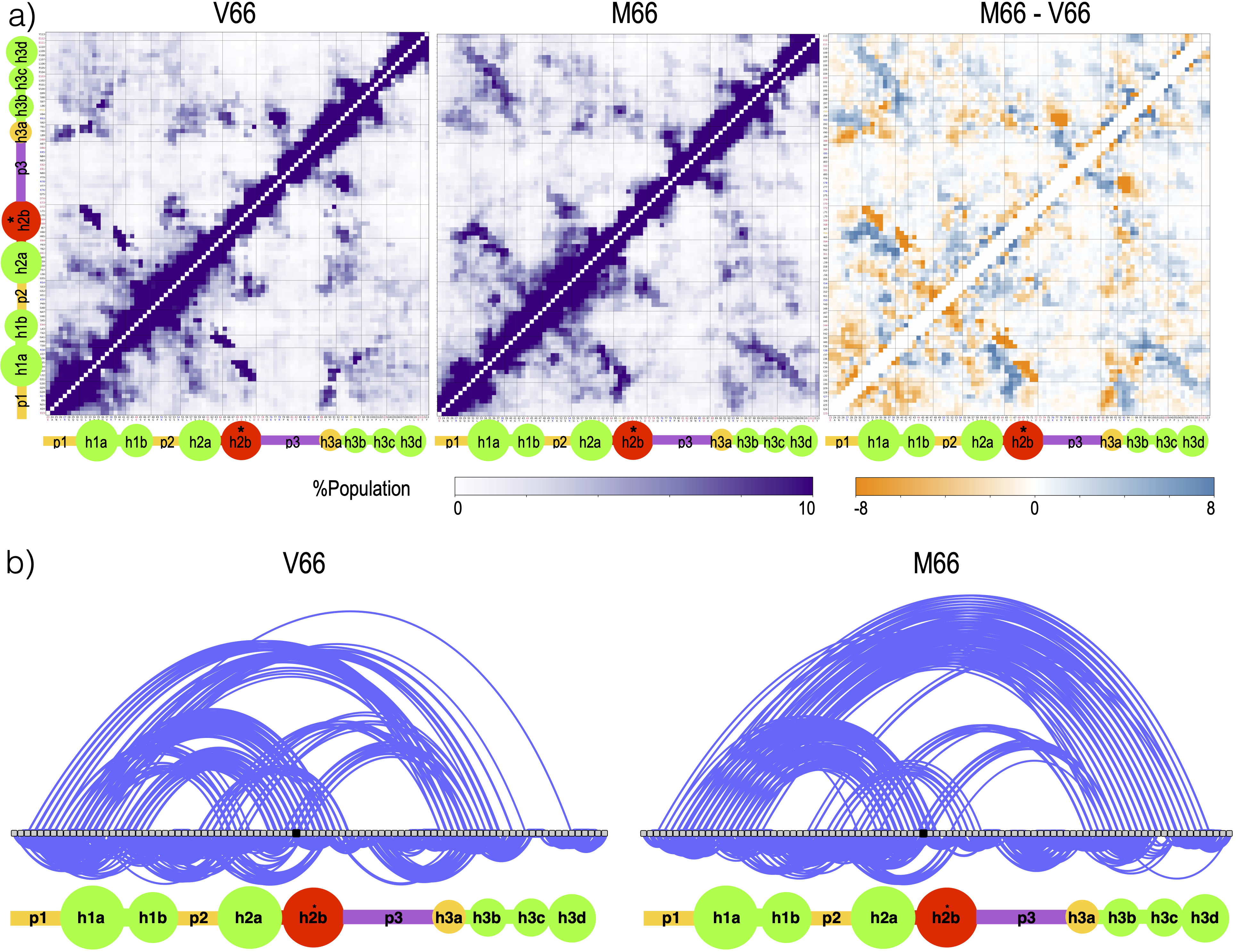

Supplement: S3 Fig — a) Contact probability between every residue pair for V66 (left), M66 (middle) sequences and the difference between the two (right). Two residue pairs are considered to be in contact if the Cα-Cα distance between the two residues is less than or equal to 0.8 nm. Panels are annotated by a blob representation of the prodomain, as in Fig 1e(i); vertical and horizontal grey lines in each panel represent the blob boundaries. b) A linear network of transient tertiary contacts shown in panel a. The contact networks were build using Cytoscape [110] with a linear representation of residues. Each protein residue comprises a node in the network, with interactions between residues represented as edges. The strength of individual interactions can be interpreted by the thickness of the edge line on the network diagram. If the separation between residues forming the contact is more than 20, its edge is drawn above the node; otherwise, the edge is drawn at the bottom of the node. To focus on significant interactions, interactions showing more than 6% persistence were considered in the network visualization. The x axis is annotated with blob representation of the prodomain, as in Fig 1e(i). (TIFF) [file pcbi.1007390.s005.tiff]

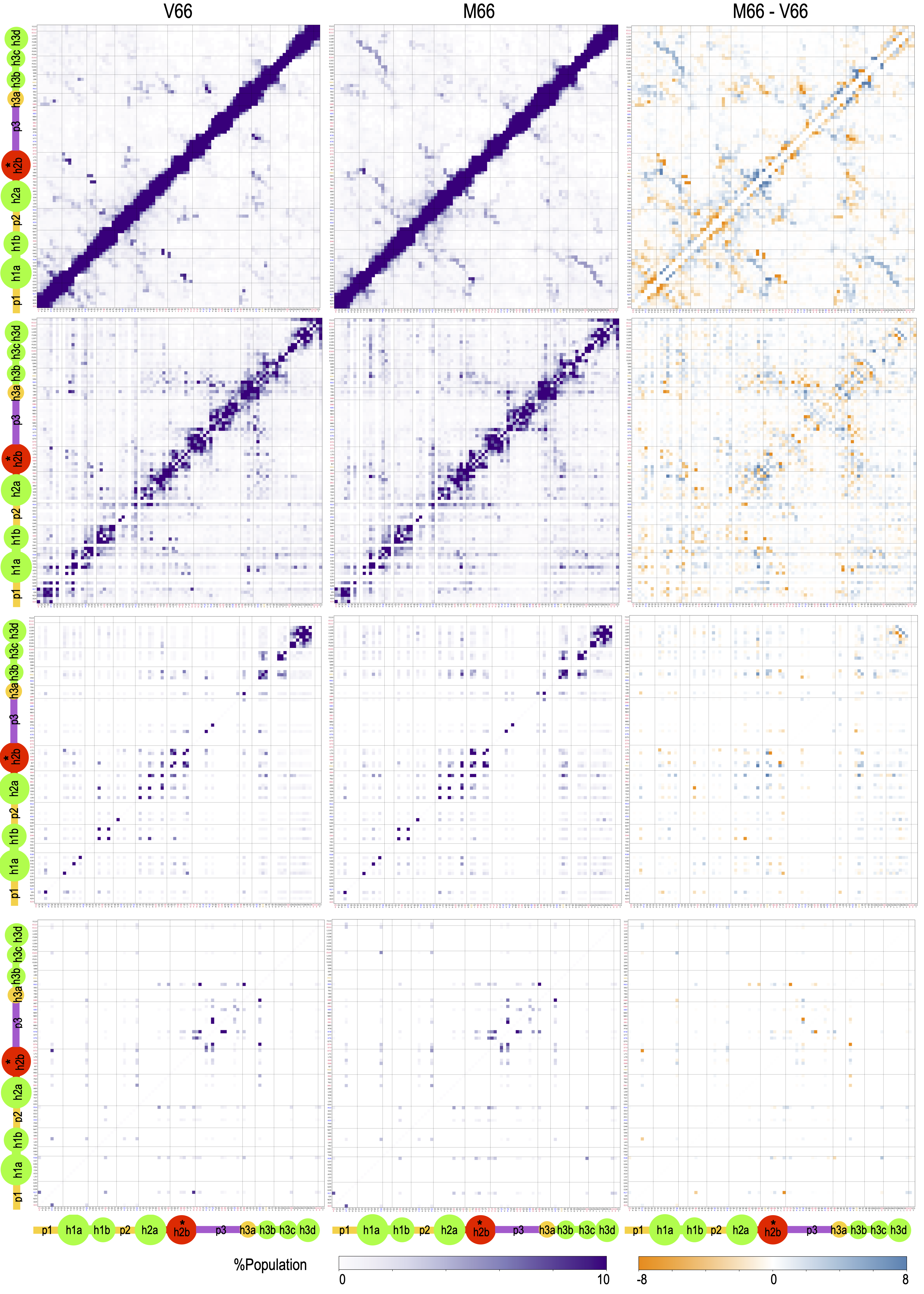

Supplement: S4 Fig — Contact probability between every residue pair for V66 (left) and M66 (middle) sequences and the difference between the two (right). Two residue pairs are in contact if the distance between backbone-backbone atoms between the two residues are 0.4 nm or less (1st row), if the distance between non hydrogen sidechain-sidechain atoms between the two residues are 0.4 nm or less (2nd row), if the distance between non hydrogen sidechain-sidechain atoms between the two hydrophobic residues are 0.4 nm or less (3rd row), if the two residue pairs are forming a salt-bridge with the distance between the donor and acceptor atoms < 0.32 nm (4th row). Panels are annotated by a blob representation of the prodomain, as in Fig 1e(i); vertical and horizontal grey lines in each panel represent the blob boundaries. (TIFF) [file pcbi.1007390.s006.tiff]

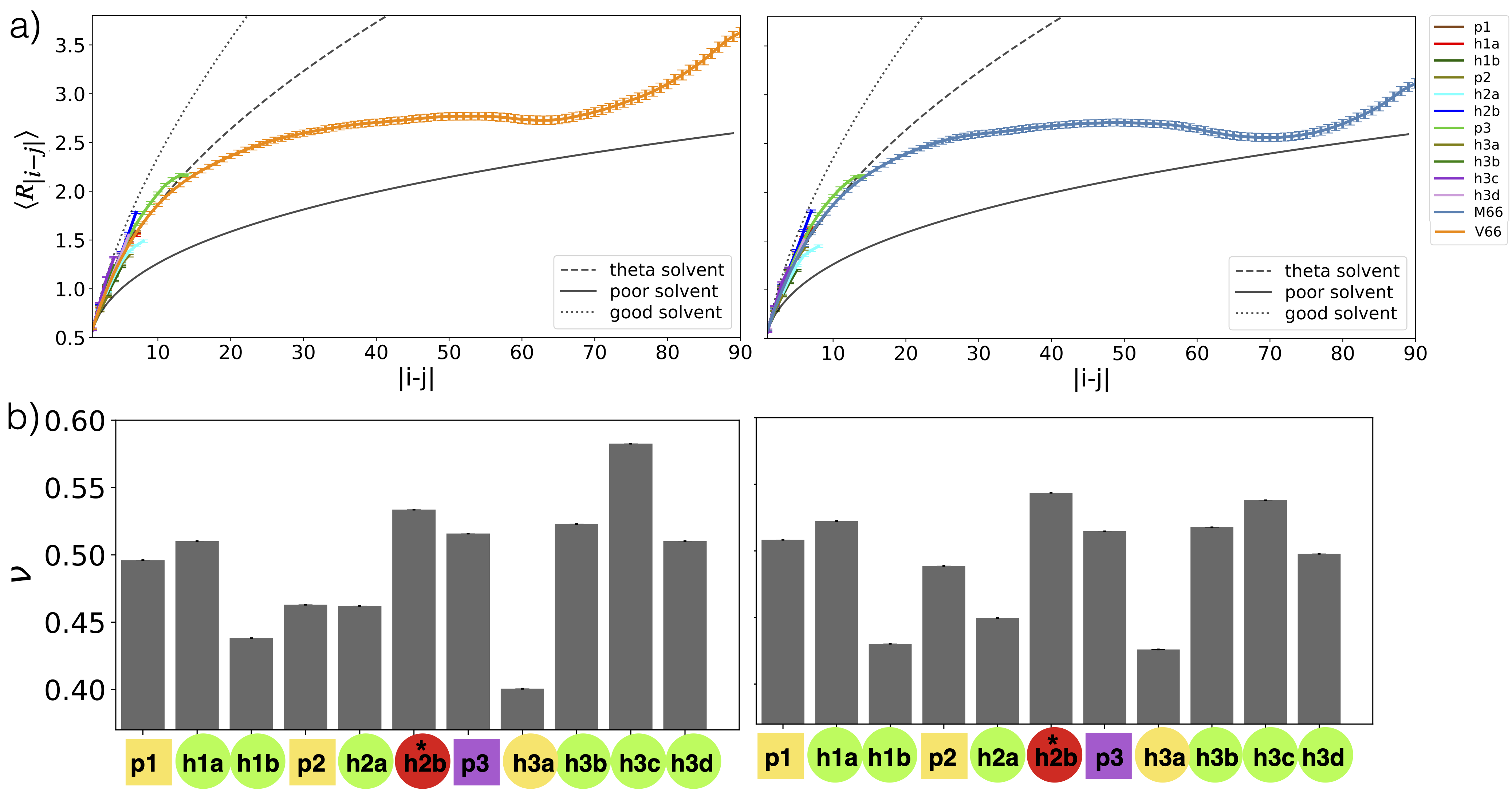

Supplement: S5 Fig — a) Mean distances between any residues i and j at 300K, for the entire V66 and M66 prodomains as well as each blob in the V66 (left) and M66 (right) sequences. Theoretical polymer scaling limits are represented by the curves 〈R|i−j|〉 = A|i − j|ν where A = 0.59 nm and ν is the Flory exponent. For good, theta, and bad solvent, ν = 3/5, 1/2, 1/3 respectively. b) Values of ν resulting from fits to each blob for V66 (left) and M66 (right) sequences. The x axis is annotated with a blob representation of the prodomain where blobs are colored according to the Das and Pappu diagram [21] in Fig 1c. (TIFF) [file pcbi.1007390.s007.tiff]

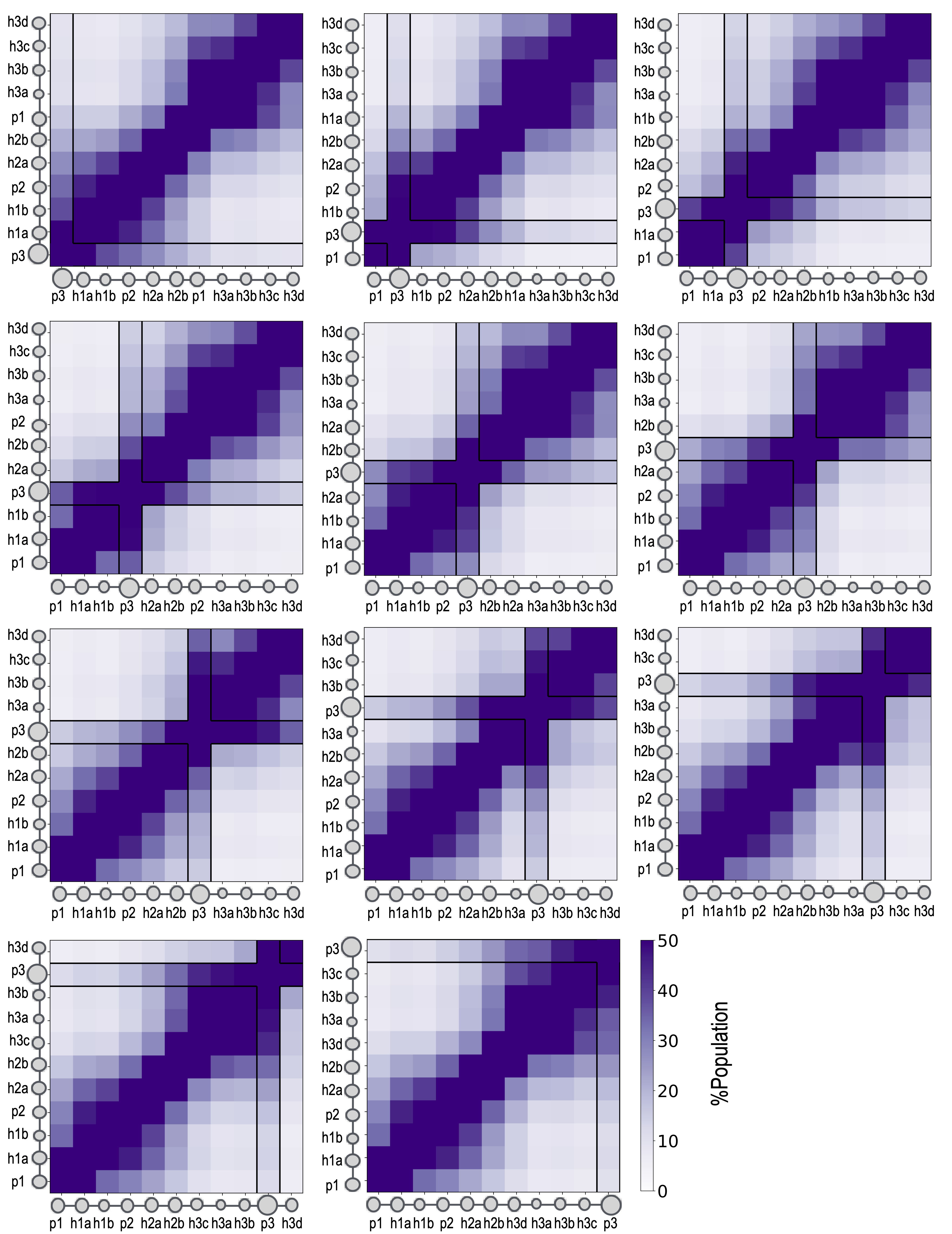

Supplement: S6 Fig — Contact probability maps from SAHP calculations, analogous to those in Fig 5a of the main text. The x and y axes are annotated with cartoon representation of the prodomain; circles are drawn to the scale of each blob’s size. Here the SAHP model is varied systematically by swapping the p3 blob with every other blob in the chain. As the p3 blob is shifted along the chain, p3 and p1 consistently bound a white “forbidden” region that has little interaction with the rest of the protein. (TIFF) [file pcbi.1007390.s008.tiff]
